# PACAP attenuates diabetic neuropathic pain by modulating ERK-PGC1α- associated mitochondrial homeostasis

**DOI:** 10.1016/j.neurot.2026.e01112

**Published:** 2026-11-07

**Authors:** Di Cao, Qingping Zhang, Shuqi Shi, Liyan Jiang, Baiyan Gong, Shaomin Yang, Zhenhui Luo, Shiwen Guo, Lizu Xiao, Mingzhu Zhai, Wuping Sun

**Affiliations:** a Department of Pain Medicine, Shenzhen Nanshan People's Hospital, Affiliated Nanshan Hospital of Shenzhen University, Shenzhen, 518060, China; b Department of Neurosurgery, Shenzhen Nanshan People's Hospital, Affiliated Nanshan Hospital of Shenzhen University, Shenzhen, 518060, China; c Department of Neurosurgery, The First Affiliated Hospital of Xi'an Jiaotong University, Xi'an, 710061, China; d Department of Clinical Laboratory Medicine, Fifth Affiliated Hospital, Southern Medical University, Guangzhou, 510900, China; e Center for Medical Experiments (CME), Shenzhen University of Advanced Technology General Hospital, Shenzhen, 518106, China; f State Key Laboratory of Bioactive Molecules and Druggability Assessment, Jinan University, Guangzhou, China

**Keywords:** Pituitary adenylate cyclase-activating polypeptide, Diabetic neuropathic pain, Mitochondrial homeostasis, PGC1α, ERK signaling

## Abstract

Diabetic neuropathic pain is a debilitating complication of diabetes for which disease-modifying therapies remain limited. Pituitary adenylate cyclase-activating polypeptide (PACAP) has been implicated in neuroprotection and metabolic regulation, but its role in diabetic neuropathic pain remains unclear. Here, we found that patients with diabetic neuropathic pain had reduced serum PACAP-38 levels and elevated pro-inflammatory cytokines compared with patients with diabetes mellitus alone. In streptozotocin-induced diabetic neuropathic pain (DNP) mice, PACAP partially improved body weight recovery but did not markedly reduce fasting hyperglycemia, glucose tolerance or pancreatic islet injury. Importantly, PACAP improved gait performance, attenuated mechanical, thermal, and cold hypersensitivity, and preserved intraepidermal nerve fibers. Transcriptomic and validation analyses showed that diabetic neuropathic pain was associated with endothelial impairment, hypoxia-related signaling, mitochondrial alterations, and redox imbalance in dorsal root ganglia. PACAP treatment partially modulated DNP-related transcriptional changes, including those linked to mitochondrial metabolism, oxidative stress-related processes, and neuronal function. Mechanistically, PACAP increased ERK phosphorylation and PGC1α expression, enhanced mitochondrial biogenesis-related responses, preserved mitochondrial ultrastructure, and improved mitochondrial membrane potential. ERK inhibition with U0126 attenuated PACAP-mediated mitochondrial protection and analgesic effects. These findings suggest that PACAP alleviates DNP through an ERK-PGC1α-associated mechanism that contributes to the restoration of mitochondrial homeostasis in sensory neurons, rather than by substantially correcting systemic hyperglycemia.

## Introduction

Diabetic peripheral neuropathy (DPN) is one of the most common chronic complications of diabetes mellitus and represents a major cause of diabetes-related morbidity. Distal symmetric polyneuropathy is the predominant clinical phenotype of DPN and may affect up to half of individuals with diabetes during the course of the disease [1], [2]. Painful diabetic peripheral neuropathy, also referred to as diabetic neuropathic pain (DNP), is a particularly debilitating manifestation characterized by chronic spontaneous pain, evoked pain, and sensory abnormalities that substantially impair quality of life [3], [4], [5]. Clinically, DNP typically presents as bilateral and symmetric distal sensory dysfunction in a length-dependent “stocking-glove” distribution, with symptoms including numbness, tingling, burning pain, allodynia, hyperalgesia, and reduced perception of pain, temperature, and vibration [1], [3]. Current clinical management mainly focuses on glycemic control, risk-factor modification, foot care, and symptomatic pain relief; however, effective disease-modifying therapies remain limited, especially for patients with established painful neuropathy [1], [4], [5]. Therefore, elucidating the molecular mechanisms underlying DNP is essential for the development of novel therapeutic strategies.

The pathogenesis of DPN and DNP is multifactorial and involves metabolic, vascular, inflammatory, and neurodegenerative mechanisms [2], [6], [7]. Among these mechanisms, mitochondrial dysfunction and oxidative stress have emerged as central contributors to peripheral nerve injury in diabetes [6], [7], [8]. Persistent hyperglycemia and metabolic overload disrupt mitochondrial electron transport and enhance the production of reactive oxygen species (ROS). When ROS production exceeds endogenous antioxidant capacity, oxidative stress causes damage to cellular lipids, proteins, and mitochondrial DNA, thereby impairing neuronal survival and function [6], [7]. Peripheral sensory neurons are particularly vulnerable to mitochondrial injury because their long axons require continuous ATP supply, efficient calcium buffering, and intact axonal transport. Mitochondrial dysfunction further reduces ATP generation, disturbs intracellular calcium homeostasis, promotes axonal degeneration, and contributes to the loss of cutaneous nerve terminals [7], [8]. Importantly, damaged mitochondria can generate additional ROS, creating a self-amplifying cycle that exacerbates oxidative stress and accelerates peripheral nerve damage. These findings support the concept that targeting the mitochondrial-oxidative stress axis may represent a promising therapeutic strategy for DNP.

Peroxisome proliferator-activated receptor gamma coactivator 1α (PGC1α) is a master regulator of mitochondrial biogenesis and oxidative metabolism. Experimental evidence indicates that impaired PGC1α signaling contributes to mitochondrial degeneration in dorsal root ganglion (DRG) neurons under diabetic conditions, whereas PGC1α overexpression protects sensory neurons from high-glucose-induced oxidative stress [9]. These observations suggest that restoration of PGC1α-dependent mitochondrial biogenesis may help preserve mitochondrial function and attenuate diabetic neuropathic injury. Several signaling pathways regulating PGC1α, including AMPK, PKA, Akt, and p38 MAPK, can act through both transcriptional regulation and post-translational modification of the PGC1α protein [10]. Collier et al. demonstrated that ERK1/2 rapidly regulates PGC1α expression and downstream mitochondrial biogenesis in renal cells and mouse kidney [11]. In neuronal systems, ERK signaling has also been linked to PGC1α regulation. BDNF was shown to stimulate PGC1α-dependent mitochondrial biogenesis in hippocampal neurons through ERK and CREB signaling [12], while ERK inhibition was reported to attenuate PGC1α upregulation in cortical neurons subjected to oxygen-glucose deprivation [13]. Collectively, these findings indicate that ERK signaling can regulate PGC1α-associated mitochondrial responses in a context-dependent manner. However, the upstream signaling mechanisms that regulate this protective mitochondrial program in DNP remain incompletely defined.

Pituitary adenylate cyclase-activating polypeptide (PACAP) is an evolutionarily conserved neuropeptide that exists mainly as two biologically active forms, PACAP-38 and PACAP-27, with PACAP-38 being the predominant endogenous form in mammals [14]. PACAP exerts its biological actions through three G protein-coupled receptors: PAC1, VPAC1, and VPAC2. Among these receptors, PAC1 displays the highest selectivity and affinity for PACAP and is widely expressed in the nervous system, including sensory pathways involved in nociception [14], [15]. Activation of PAC1 can stimulate adenylyl cyclase, increase cyclic adenosine monophosphate (cAMP) levels, and activate downstream signaling pathways such as protein kinase A (PKA) and extracellular signal-regulated kinase (ERK) [14], [15]. Several studies have demonstrated that PACAP can activate ERK signaling in neuronal and neuroendocrine systems [16], [17]. PACAP has been widely reported to exert neuroprotective effects through anti-apoptotic, anti-inflammatory, antioxidant, and mitochondrial protective mechanisms [14], [18], [19], [20], [21]. In experimental diabetic neuropathy, PACAP treatment has been shown to attenuate mechanical hyperalgesia and reduce structural abnormalities, including axon–myelin separation, mitochondrial fragmentation, unmyelinated fiber atrophy, and endoneurial vascular changes [22]. Importantly, PACAP has also been implicated in the regulation of PGC1α-associated mitochondrial function. PACAP treatment increased mitochondrial activity and PGC1α expression in neuronal models, whereas PGC1α knockdown attenuated PACAP-associated mitochondrial activation and neurite outgrowth [23]. Given the reported roles of PACAP in ERK regulation and PGC1α-associated mitochondrial function, whether this signaling relationship contributes to the protective effects of PACAP in DNP remains unclear.

In the present study, we found that patients with diabetic neuropathy exhibited significantly lower serum PACAP-38 levels than patients with diabetes mellitus alone, accompanied by markedly elevated levels of proinflammatory cytokines, including interferon-γ (IFN-γ), interleukin-17A (IL-17A), and tumor necrosis factor-α (TNF-α). To further investigate the functional role of PACAP in DNP, we established a streptozotocin-induced DNP mouse model and administered exogenous PACAP-38 as an intervention. PACAP-38 treatment significantly alleviated pain hypersensitivity and reduced the loss of cutaneous nerve terminals in diabetic mice. Mechanistically, PACAP-38 was associated with increased ERK phosphorylation and PGC1α expression, accompanied by improved mitochondrial morphology and function and reduced oxidative stress in the DRG. Pharmacological inhibition of ERK signaling attenuated several of these PACAP-associated protective effects, including the preservation of mitochondrial phenotypes and PGC1α expression. Together, these findings suggest that PACAP-38 exerts neuroprotective and analgesic effects in DNP and support a potential contribution of MEK/ERK signaling to PACAP-mediated mitochondrial protection and PGC1α-associated responses.

## Materials and methods

### Clinical specimen acquisition

This cross-sectional study was approved by the Ethics Committee of the Fifth Affiliated Hospital of Southern Medical University (Approval No. [2025-JYYXK-J-002]) and conducted in accordance with the Declaration of Helsinki. Written informed consent was obtained from all participants prior to enrollment. The study enrolled age-matched healthy controls, patients with type 2 diabetes mellitus (T2DM) without neuropathy, and patients with T2DM complicated by DNP diagnosed according to the Toronto Consensus Criteria. A total of 69 participants met all eligibility criteria and completed the full study protocol. All diabetic participants underwent standardized clinical evaluation and nerve conduction studies (NCS), whereas healthy controls did not undergo NCS. Fasting venous blood samples were collected, and sera were isolated. Serum levels of interferon-gamma (IFN-γ), interleukin-2 (IL-2), IL-4, IL-6, IL-10, IL-17A, and tumor necrosis factor-alpha (TNF-α) were measured using a fully automated chemiluminescence immunoassay kit. Serum concentration of pituitary adenylate cyclase-activating polypeptide-38 (PACAP-38) was determined by enzyme-linked immunosorbent assay (Finetest, EH3494).

### Animals

All animal experiments were reviewed and approved by the Institutional Animal Care and Use Committee (IACUC) of Nanshan People's Hospital, Shenzhen (approval No. NSHP-JY-25015), and were performed in accordance with institutional guidelines for the care and use of laboratory animals. Six-week-old adult male C57BL/6 mice were purchased from the Guangdong Provincial Medical Experimental Animal Center and housed under standard laboratory conditions with a 12 h light/dark cycle and free access to food and water.

### Mouse DNP model

The diabetic neuropathy model was induced by five consecutive daily intraperitoneal injections of streptozotocin (STZ; Sigma-Aldrich, S0130) at a cumulative dose of 200 mg/kg body weight. STZ was freshly dissolved in 0.1 mol/L sodium citrate buffer (pH 4.2–4.5) on ice in the dark and administered immediately after preparation. Mice were fasted for 12 h before injection but had free access to water. Mice receiving an equivalent volume of citrate buffer instead of STZ were designated as the Mock group and served as non-diabetic vehicle controls. One week after the final injection, diabetes was confirmed by measuring tail-vein blood glucose levels. Only mice with blood glucose levels >16.7 mmol/L were included in the subsequent experiments. The day of diabetes confirmation was designated as day 0 of drug treatment. Mice in the PACAP group received PACAP-38 (0.1 mg/kg; MCE, HY-P0221), whereas mice in the PACAP-38 + U0126 group received PACAP-38 (0.1 mg/kg) together with the MEK/ERK inhibitor U0126 (5 mg/kg; Selleck, S1102). Both agents were administered via intraperitoneal injection every other day from day 0 to day 28. Body weight, blood glucose levels, and behavioral tests were measured on days 0, 1, 3, 5, 7, 14, 21, and 28 after the initiation of drug administration, and an intraperitoneal glucose tolerance test (IPGTT) was performed on day 30.

### Behavioral tests

Before baseline behavioral testing, mice were acclimated to the testing environment for 1–2 h per day for 3 consecutive days. Mechanical, cold, and thermal sensitivity tests were performed with a 1 h interval between assays. Mechanical paw withdrawal thresholds were measured using the up-down method as previously described [24], [25]. Briefly, mice were placed in transparent chambers on an elevated wire grid and habituated for 30 min before testing. A 0.16 g von Frey filament was applied to the midplantar surface of the hind paw until it bent into a “C” shape and held in place for 6–8 s. If the mouse did not withdraw its paw, the next stronger filament was used; if a withdrawal response occurred, the next weaker filament was applied. This sequence was repeated four times after each positive response. The 50% paw withdrawal threshold was calculated using the formula: (g) = 10ˆ(Xf + kδ)/10,000.

Cold sensitivity was assessed using a cold plate test at 4 °C as previously reported [26], [27]. Each mouse was placed in a glass chamber on the cold plate, and paw withdrawal latency was defined as the time from hind paw contact with the plate to paw licking or withdrawal. Each paw was tested in triplicate at 10 min intervals. A cutoff time of 60 s was used to prevent tissue damage. Thermal sensitivity was evaluated using the same protocol, except that the plate temperature was maintained at 52 °C.

### Gait recording and analysis

The gait of mice was recorded and analyzed via the Rodent Gait Analysis System (SA114, SansBio, China). To minimize stress, mice were habituated to the testing environment before the formal experiment. During testing, each mouse was allowed to walk freely along a glass walkway. Footprint images were captured by a camera positioned beneath the walkway, with contrast enhanced by a green fluorescent backlight. Trials were repeated until three consecutive, uninterrupted walking sequences were recorded for each animal. Gait parameters, including step pattern coordination, brake index, instantaneous velocity, paw contact duration and area, were calculated with the system software and subsequently analyzed.

### Cell culture and OGD treatment

SH-SY5Y cells were cultured in Dulbecco's Modified Eagle Medium (DMEM; Cytiva, SH30022.01) supplemented with 10% fetal bovine serum (FBS; Excell, FSP500) and 1% penicillin-streptomycin (Gibco, 15140122) at 37 °C in a humidified incubator with 95% O_2_ and 5% CO_2_. Oxygen-glucose deprivation (OGD) model was used to mimic hypoxic and glucose-deprived stress associated with the microenvironmental alterations observed in DNP DRGs [28]. Briefly, the culture medium was replaced with glucose-free DMEM (Procell, PM150278), and cells were immediately transferred to a hypoxia chamber (Billups-rothenberg, MIC-101). The chamber was flushed with a gas mixture consisting of 94% N_2_, 5% CO_2_, and 1% O_2_ to displace ambient air, and then sealed and placed in a standard cell culture incubator at 37 °C for the desired duration to mimic concurrent hypoxia and glucose deprivation.

To determine an appropriate duration of OGD for subsequent experiments, SH-SY5Y cells were exposed to OGD for 1, 3, or 5 h, followed by assessment of oxidative stress and mitochondrial membrane potential using ROS and JC-1 assays, respectively. Based on these preliminary experiments, 3 h OGD was selected for subsequent studies. For PACAP-38 treatment, PACAP-38 was added to glucose-free medium immediately before OGD initiation and remained present throughout the 3 h OGD exposure. For MEK/ERK inhibition experiments, PACAP-38 and U0126 were added simultaneously immediately before OGD initiation and remained present throughout the 3 h OGD exposure. Following OGD, cells were harvested for assessment of mitochondrial function and relevant molecular markers.

### DRG primary neuronal culture

Dorsal root ganglia (DRG) were isolated from 4∼6-week-old C57BL/6 mice under aseptic conditions. Tissues were digested with collagenase (Sigma, C0130; 0.4 mg/mL), DNase I (Macklin, D875698; 0.1 mg/mL), and trypsin (Sigma, T8003; 1 mg/mL) for 30 min. After washing and low-speed centrifugation, cells were resuspended in neurobasal medium supplemented with B27 and plated onto poly-D-lysine coated glass coverslips. Cells were then cultured in a humidified incubator at 37 °C with 5% CO_2_.

### RNA extraction and quantitative real-time PCR (RT-qPCR)

Total RNA was extracted from cells or mouse tissues using TRIzol reagent (Invitrogen, 15596018CN), and cDNA was synthesized using a cDNA Synthesis Kit (SparkJade, AG0305) according to the manufacturer's instructions. Quantitative real-time PCR was performed using SYBR qPCR Mix (Genstar, A304) on a real-time PCR instrument. β-actin was used as the housekeeping gene. Primer sequences are listed in Table 1.

**Table 1:** List of qPCR primers (m stands for mouse; h stands for human).

Primer name	Sequence (5′-3)
*m*-PGC1α	F: CCCAAGGGTTCCCCATTTGA; R: GCAAGAGGGCTTCAGCTTTG
h-PGC1α	F: GAAAAATAGGAGCCGGGAAT R: CCGAAGAGTTGCTGCAGTTT
*m*–HIF–1α	F: GATGGCTTTGTGATGGTGCTAAC; R: CTCCGCTGTGTGTTTAGTTCTTT
h- HIF-1α	F: AGACAAAGTTCACCTGAGCCTAAT R: GGGTTCTTTGCTTCTGTGTCTTC
*m*-RPL13A	F: GGGCAGGTTCTGGTATTGGAT; R: GGCTCGGAAATGGTAGGGG
m-Cytochrome b	F: TGCATACGCCATTCTACGCT; R: AGGCTTCGTTGCTTTGAGGT
m-β-actin	F: CTAGGCGGACTGTTACTGAGC; R: ATGTTTGCTCCAACCAACTGC
h-β-actin	F: CCTTCCTGGGCATGGAGTC R: TGATCTTCATTGTGCTGGGTG

### Transmission electron microscopy (TEM)

DRG tissues were rapidly excised from C57BL/6 mice and fixed in electron microscopy fixative (Servicebio, G1102) for 2 h at room temperature, followed by storage and transport at 4 °C. After dehydration through a graded ethanol series, tissues were embedded in epoxy resin and sectioned into ultrathin slices. Sections were stained with uranyl acetate and lead citrate, and mitochondrial morphology and number were examined using a transmission electron microscope (Hitachi, HT7800).

### Femtosecond laser label-free imaging (FLl)

Dorsal root ganglia (DRG) tissues were imaged using a label-free multimodal imaging system (FI-100; Guangzhou Femto Research Center). This system delivers sub-5 fs ultrashort laser pulses via a high-numerical-aperture objective (Olympus UAPON 40 × W340, NA 1.15) without requiring tissue sectioning or exogenous staining. The system synchronously acquires third-harmonic generation (THG), third-photon excitation fluorescence (3PEF), and second-harmonic generation (SHG) signals. Specifically, the 3PEF channel captures the intrinsic fluorescence of metabolic cofactors NAD(P)H and FAD. Since the fluorescence intensity of these endogenous fluorophores correlates with their respective pool concentrations in the cellular redox state, this approach enables quantitative, label-free metabolic imaging of DRG microstructures.

### Western blot

SH-SY5Y cells or mouse tissues were lysed in RIPA lysis buffer (Beyotime, P0038) supplemented with protease and phosphatase inhibitors. Equal amounts of protein were separated by SDS-PAGE and transferred onto PVDF membranes (Merck, ISEQ00010). Membranes were blocked with blocking buffer (Beyotime, P0023B) for 30 min and incubated with primary antibodies overnight at 4 °C. After washing with TBST, membranes were incubated with HRP-conjugated secondary antibodies for 1 h at room temperature. Protein bands were detected using enhanced chemiluminescence (ECL) reagent (Beyotime, P0018) and visualized with a bioimaging system. Band intensities were quantified using ImageJ software. Antibodies used for Western blotting are listed in Table 2.

**Table 2:** List of protein antibodies.

Protein	Antibodies name	Brand	Cat no.
PGC1α	PGC1α rabbit pAb	ABclonal	A12348
HIF-1α	Anti–HIF–1 alpha antibody	Abcam	Ab179483
TNFα	TNF alpha antibody	Affbiotech	AF7014
Tom20	TOM20 polyclonal antibody	Proteintech	11802-1-AP
PAC1	ADCYAP1R1 antibody	CUSABIO	CSB-PA207627
BAX	BAX polyclonal antibody	Proteintech	50599-2-Ig
Bcl-2	Anti-Bcl-2 antibody	Abcam	Ab182858
CD31	Human/Mouse/Rat CD31/PECAM-1 antibody	R&D systems	AF3628
PGP9.5	Anti-PGP9.5 antibody - neuronal marker	Abcam	Ab108986
*p*-ERK	Phospho-p44/42 MAPK (Erk1/2) (Thr202/Tyr204) rabbit monoclonal antibody	Cell signaling technology	4370 T
ERK	p44/42 MAPK (Erk1/2) (137F5) rabbit monoclonal antibody	Cell signaling technology	4695 T
*p*-JNK	Phospho-SAPK/JNK (Thr183/Tyr185) rabbit monoclonal antibody	Cell signaling technology	4671 T
JNK	JNK monoclonal antibody	Proteintech	66210-1-Ig
p-p38	Phospho-p38 MAPK (Thr180/Tyr182) rabbit monoclonal antibody	Cell signaling technology	9215 T
P38	p38 MAPK monoclonal antibody	Proteintech	14064-1-AP

### ELISA

Serum samples from humans were analyzed according to the manufacturer's instructions (FineTest, EH3494). Briefly, diluted standards and serum samples were added to microplate wells, sealed, and incubated at 37 °C for 90 min. After washing, biotin-antibody working solution was added and incubated at 37 °C for 60 min, followed by HRP-streptavidin (SABC) working solution at 37 °C for 30 min. After five washes, chromogenic substrate was added and incubated at 37 °C for 20 min. The reaction was stopped with stop solution, and absorbance was measured immediately at 450 nm.

### Immunofluorescence staining

Fresh tissues were fixed in 4% paraformaldehyde, cryoprotected in 20% and 30% sucrose solutions, and sectioned at 15 μm. Sections were blocked with blocking buffer (Beyotime, P0260) for 30 min and incubated with primary antibodies overnight at 4 °C. The next day, after three washes with TBST, sections were incubated with fluorescent secondary antibodies for 2 h at room temperature. After three additional washes, sections were mounted with mounting medium (Servicebio, G1407) and imaged using a laser scanning confocal microscope (Olympus).

### Intracellular ROS, superoxide, and MDA assay

Detection of intracellular ROS (Beyotime, S0033), superoxide anions (Beyotime, S0061) and Malondialdehyde (MDA) (Beyotime, S0131) was performed according to the manufacturer's instructions. Briefly, probes were loaded into cells 3 h after the establishment of the OGD model, followed by observation at specific wavelengths.

### Intracellular ATP assay

After cells were subjected to OGD and drug treatment, they were lysed and centrifuged at 12,000 rpm at 4 °C for 5 min. The supernatant was collected for the determination of adenosine triphosphate (ATP) using an ATP assay kit (Beyotime, S0027) according to the manufacturer's instructions.

### Mitochondrial membrane potential assay

Mitochondrial membrane potential (ΔΨm) was assessed using the JC-1 fluorescent probe (Beyotime, C2003). Cells were incubated with JC-1 staining working solution according to the manufacturer's instructions for 20 min at 37 °C, washed with JC-1 buffer, and then maintained in culture medium for imaging by confocal microscopy.

### RNA-seq analysis and gene set enrichment analysis (GSEA)

Total RNA was isolated from the L4–L6 DRG tissues of mice in the control, DNP, and DNP + PACAP-38 treatment groups using TRIzol reagent. High-throughput RNA sequencing was performed by TsingKe biotech Co., Ltd. (Beijing, China) following the manufacturer's instructions. After normalization, differentially expressed genes (DEGs) were identified using the CytoNavigator™ RNA-seq data analysis system, with a threshold of absolute fold change (FC) > 2 and a false discovery rate (FDR) < 0.05 considered statistically significant. Functional enrichment analyses, including Gene Ontology (GO), Kyoto Encyclopedia of Genes and Genomes (KEGG) pathway, Gene Set Enrichment Analysis (GSEA), and Series Cluster trend analysis, were subsequently performed.

### Data and materials availability

All data supporting the results of this study are available within the paper, Supplementary Information and Source Data, where raw values, exact *P*-values and gel images are included. Researchers may contact the corresponding author to request additional data. The raw RNA-seq data generated from this study are accessible in the NCBI SRA database under BioProject PRJNA1474944 and PRJNA1483870.

### Statistical analysis

All data were analyzed using GraphPad Prism 10.0 software. Unpaired or paired Student's *t*-test was applied for comparisons between two groups. One-way ANOVA followed by Tukey's post-hoc test was used for multiple-group comparisons with a single factor, while two-way ANOVA combined with Bonferroni's post-hoc test was adopted for data with two independent variables. Data are shown as mean ± standard error mean (SEM), and statistical significance is denoted as follows: NS, not significant; ∗*P* < 0.05; ∗∗*P* < 0.01; ∗∗∗*P* < 0.001; ∗∗∗∗*P* < 0.0001.

## Results

### Elevated pro-inflammatory cytokines and reduced PACAP-38 are associated with peripheral nerve dysfunction in DNP

A total of 26 healthy controls, 26 patients with type 2 diabetes mellitus (T2DM), and 26 patients with DNP were included in this study. Baseline clinical analysis showed that patients in the DNP group had a longer duration of diabetes than those in the T2DM group (Table 3). Fasting blood glucose and glycated hemoglobin (HbA1c) levels were significantly increased in both the T2DM and DNP groups compared with healthy controls, with the highest levels observed in patients with DNP (Fig. 1A and B). Peripheral nerve conduction studies revealed marked neurophysiological abnormalities in the DNP group. Compared with T2DM patients, patients with DNP showed significantly reduced bilateral sural nerve conduction velocities, prolonged nerve conduction latencies, and decreased nerve action potential amplitudes (Fig. 1C–F, Fig. S1A–C). These findings indicate substantial peripheral nerve dysfunction in DNP, involving both demyelinating changes and axonal injury.

**Table 3:** Baseline demographic and clinical-biochemical characteristics of study participants.

Variable	Healthy (n = 26)	T2DM (n = 26)	DNP (n = 26)	P Value		
				Healthy *vs* T2DM	Healthy *vs* DNP	T2DM *vs* DNP
Male [n (%)]	13 (50%)	12 (46%)	15 (58%)	–	–	–
Female [n (%)]	13 (50%)	14 (54%)	11 (42%)	–	–	–
Age (y/o)	58.50 ± 1.05	65.08 ± 2.13	61.27 ± 1.87	–	–	–
BMI	–	22.39 ± 0.84	23.90 ± 0.96	–	–	0.9638
Course (years)	–	8.92 ± 1.34	13.00 ± 1.84	–	–	0.0796
Glucose (mmol/L)	4.89 ± 0.10	11.12 ± 1.13	12.88 ± 1.28	P < 0.0001	P < 0.0001	0.4208
HbA1c (%)	5.46 ± 0.06	8.17 ± 0.60	9.37 ± 0.53	0.0014	P < 0.0001	0.1785
TC (mmol/L)	4.44 ± 0.15	4.73 ± 0.24	4.35 ± 0.22	0.6063	0.9606	0.4358
TG (mmol/L)	1.07 ± 0.07	2.77 ± 0.59	2.19 ± 0.36	0.0119	0.1247	0.5799
HDL-C (mmol/L)	1.47 ± 0.07	1.10 ± 0.09	1.15 ± 0.08	0.0069	0.0181	0.9243
LDL-C (mmol/L)	2.70 ± 0.13	3.04 ± 0.21	2.66 ± 0.17	0.3731	0.9845	0.2827

**Footnotes**: Overall group-comparison P-value by one-way ANOVA. Continuous data are presented as mean ± SEM; categorical data are shown as counts and percentages. TC, total cholesterol, reference range: 0–5.17 mmol/L; TG, triglyceride, reference range: 0.7–1.70 mmol/L; HDL-C, high-density-lipoprotein cholesterol, reference range: 1.03–1.54 mmol/L; LDL-C, low-density-lipoprotein cholesterol, reference range: 0–3.37 mmol/L.

**Fig. 1:**
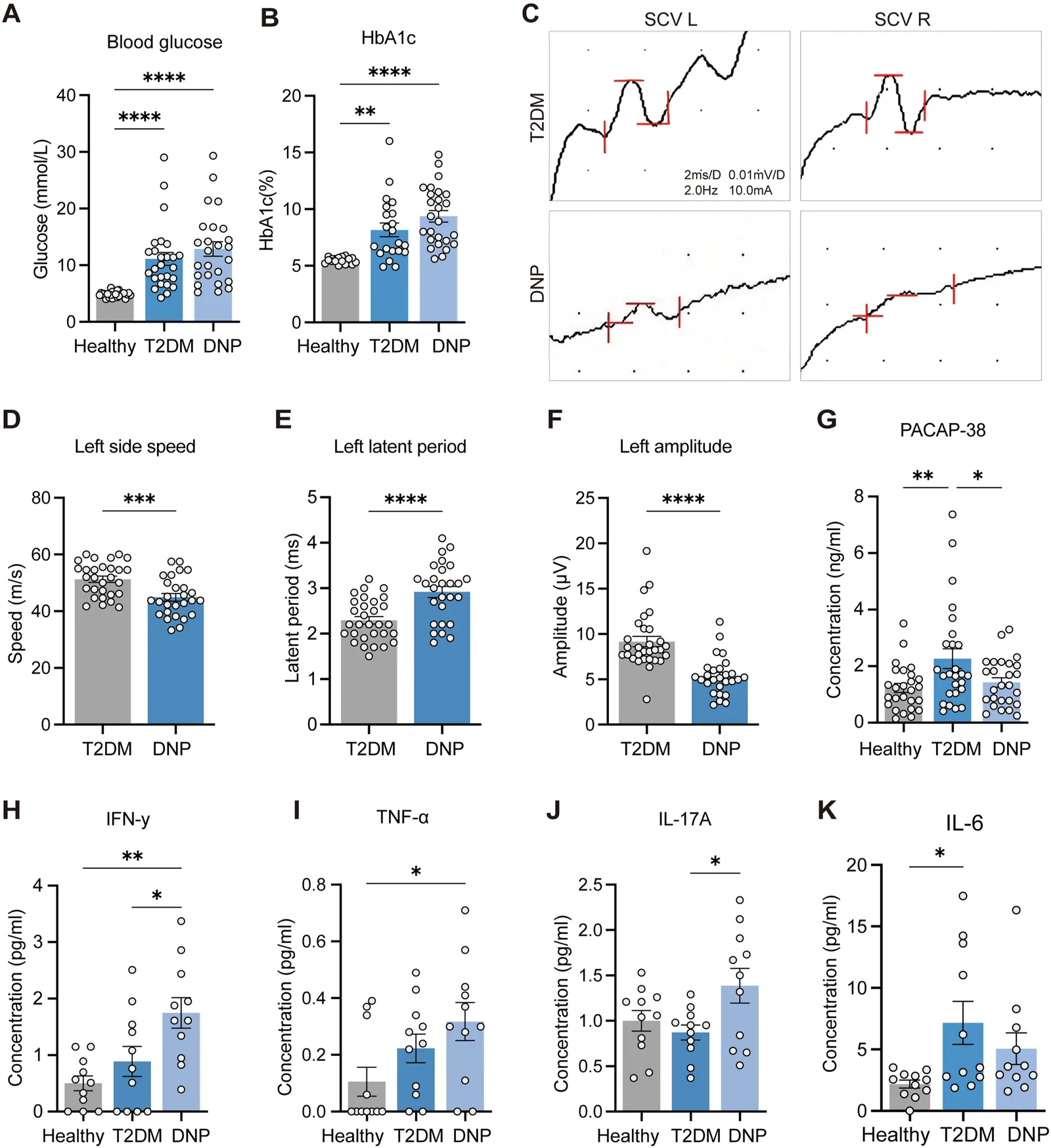
Clinical, neurophysiological, and immunological characteristics of study participants.**(A**–**B)** Quantification of fasting blood glucose (A) and glycated hemoglobin (HbA1c) (B) levels across the three groups. **(C)** Representative waveforms of the sural sensory nerve action potential (SNAP) recorded from the left (SCV L) and right (SCV R) sural nerves in T2DM and DNP patients. Red brackets indicate the measurement points for latency and amplitude. **(D**–**F)** Quantitative analysis of neurophysiological parameters: (D) sensory nerve conduction velocity, (E) distal latency, and (F) SNAP amplitude for the left sural nerves in T2DM and DNP patients. **(G**–**K)** Serum concentrations of neuropeptides and inflammatory cytokines, including PACAP-38 (G), IFN-γ (H), TNF-α (I), IL-17A (J) and IL-6 (K) in healthy controls, T2DM, and DNP patients. Data are presented as the mean ± SEM. Individual data points are shown. Statistical significance was assessed using an unpaired two-tailed Student's *t*-test for comparisons between T2DM and DNP groups and one-way ANOVA followed by Tukey's post hoc test for comparisons among the Healthy, T2DM, and DNP groups. ∗*p* < 0.05, ∗∗*p* < 0.01, ∗∗∗*p* < 0.001, ∗∗∗∗*p* < 0.0001.

Serum analysis further demonstrated distinct alterations in inflammatory mediators and neuropeptides among the three groups. Given the established roles of PACAP-38 in neuroprotection and pain modulation, we further examined circulating PACAP-38 levels in the three groups. Notably, circulating PACAP-38 levels were significantly higher in patients with T2DM than in healthy controls and significantly lower in patients with DNP than in T2DM patients, while remaining numerically higher than those in healthy controls (Fig. 1G). Sex-stratified analysis showed a similar overall pattern in males and females, with no significant sex-by-clinical group interaction (Fig. S1D). The levels of pro-inflammatory cytokines, including interferon-γ (IFN-γ), interleukin-17A (IL-17A), tumor necrosis factor-α (TNF-α) (Fig. 1H–J) increased progressively from healthy controls to patients with T2DM and were highest in patients with DNP. IFN-γ was significantly higher in DNP patients than in both healthy controls and T2DM patients, TNF-α was significantly higher in DNP patients than in healthy controls, and IL-17A was significantly higher in DNP patients than in T2DM patients. IL-6 was also significantly increased in T2DM patients compared with healthy controls (Fig. 1K). In contrast, anti-inflammatory cytokines, including IL-2, IL-10, and IL-4 (Fig. S1E–G), showed no significant differences among the groups or only minor changes. Collectively, these results indicate that DNP is associated with impaired peripheral nerve conduction, increased levels of selected pro-inflammatory cytokines, and altered circulating PACAP-38 levels.

### PACAP-38 has limited effects on metabolic phenotypes in STZ-induced DNP mice

To determine whether PACAP modulates diabetic metabolic phenotypes, we established a STZ-induced mouse model of DNP and administered PACAP-38 intervention according to the protocol outlined in Fig. 2A. Compared with the Mock group, DNP mice exhibited a significant reduction in body weight during the early phase after STZ injection. PACAP-38 treatment partially attenuated this weight loss, although a significant difference between the PACAP and DNP groups was observed only at day 14 (Fig. 2B). Notably, analysis of the longitudinal body-weight trajectory showed a significantly greater area under the curve (AUC) in PACAP-treated mice compared with DNP mice, indicating a modest overall improvement in body-weight trajectory (Fig. 2C).

**Fig. 2:**
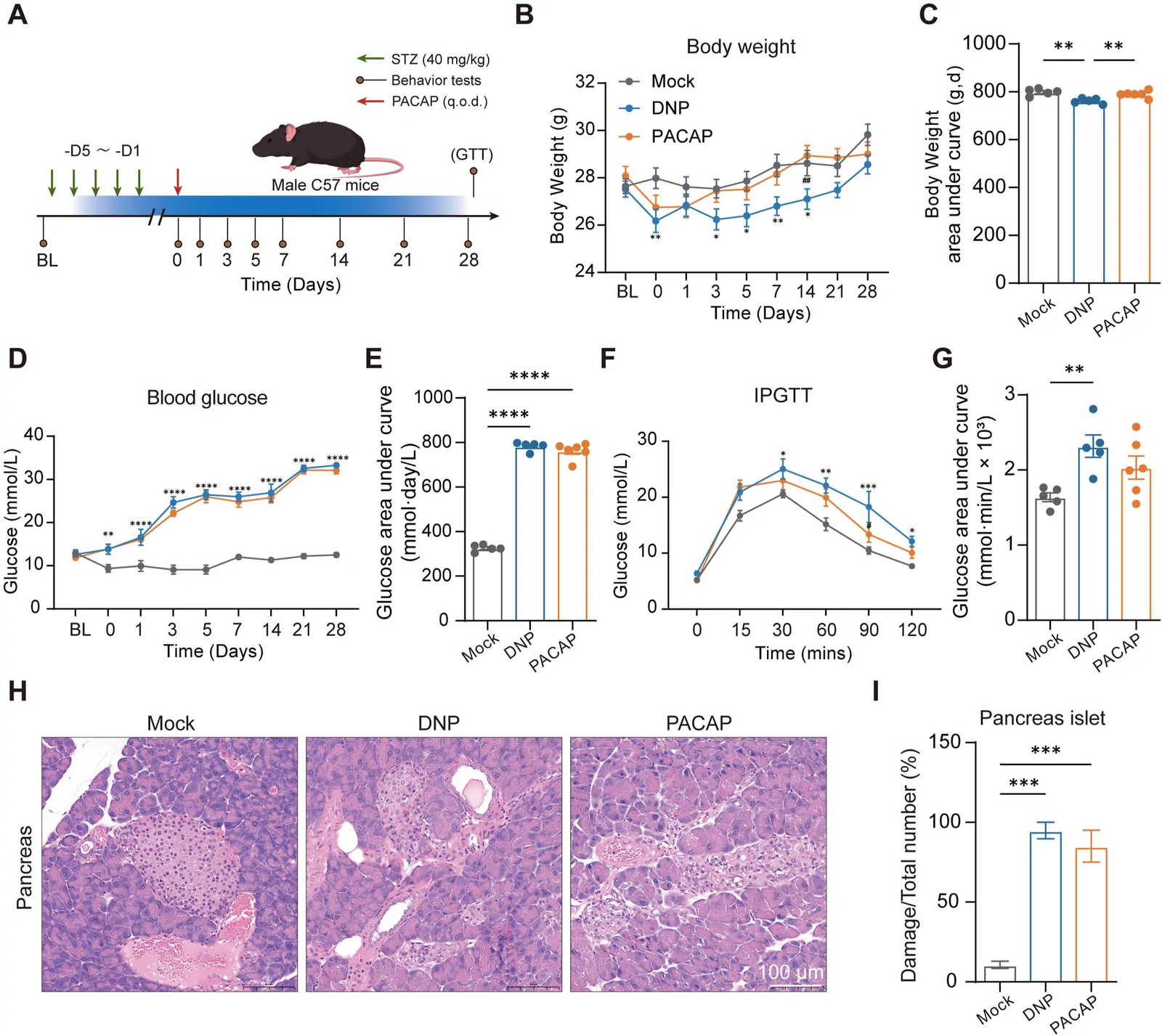
PACAP-38 has limited effects on metabolic parameters and pancreatic islet injury in STZ-induced DNP mice.**(A)** Schematic diagram of the animal experimental design. Male C57BL/6 mice were intraperitoneally injected with streptozotocin (STZ, 40 mg/kg/day) for 5 consecutive days (total 200 mg/kg) to establish the DNP model. After confirmation of diabetes, mice received intraperitoneal PACAP-38 (0.1 mg/kg, every other day). Body weight, fasting blood glucose, behavioral tests, and IPGTT were assessed at the indicated time points. Mock mice received citrate buffer instead of STZ and served as non-diabetic vehicle controls. DNP mice received STZ, and PACAP mice were STZ-induced DNP mice treated with PACAP-38. **(B, C)** Dynamic changes in body weight (B) and corresponding area under the curve (AUC) analysis (C) in Mock, DNP, and PACAP-38-treated mice. **(D, E)** Longitudinal monitoring of fasting blood glucose levels (D) and AUC analysis (E) across the three groups. **(F, G)** Blood glucose curves during the IPGTT (F) and corresponding AUC quantification (G), reflecting systemic glucose tolerance in each group. **(H, I)** Representative hematoxylin and eosin (H&E) staining images of pancreatic islets (H) and quantitative analysis of the islet damage ratio (I). Scale bar, 100 μm. Data are presented as mean ± SEM. Statistical significance was assessed using two-way repeated-measures ANOVA for longitudinal measurements and one-way ANOVA followed by Tukey's post hoc test. Asterisks indicate comparisons with the Mock group, and hash symbols indicate comparisons with the DNP group. ∗*p* < 0.05, ∗∗*p* < 0.01, ∗∗∗*p* < 0.001, ∗∗∗∗*p* < 0.0001.

Longitudinal monitoring of fasting blood glucose showed sustained hyperglycemia in DNP mice throughout the experiment, and PACAP administration did not significantly alter fasting blood glucose levels (Fig. 2D and E). To further assess systemic glucose homeostasis, an IPGTT was performed. DNP mice exhibited impaired glucose clearance following glucose challenge. PACAP-38 treatment resulted in a significantly lower glucose level at 90 min compared with untreated DNP mice, whereas the overall IPGTT AUC did not differ significantly between the two groups, despite a numerically lower AUC in PACAP-treated mice (Fig. 2F and G). Histological analysis of pancreatic tissues by hematoxylin and eosin (H&E) staining showed preserved islet architecture and minimal pathological changes in Mock mice. In contrast, DNP mice exhibited marked islet atrophy, disorganized cellular arrangement, and structural disruption. PACAP-38 intervention did not visibly ameliorate STZ-induced islet damage or reduce the proportion of damaged islets (Fig. 2H and I). Together, these findings suggest that PACAP-38 produces modest effects on body-weight trajectory but does not substantially correct fasting hyperglycemia, overall glucose tolerance, or pancreatic islet injury in STZ-induced DNP mice.

### PACAP-38 ameliorates gait dysfunction and peripheral neuropathy in STZ-induced DNP mice

We next evaluated whether PACAP ameliorates the functional and pathological manifestations of diabetic neuropathy in STZ-induced DNP mice. Dynamic gait kinematic analysis was performed to quantitatively assess locomotor function across the experimental groups (Fig. 3A). Compared with Mock mice, DNP mice exhibited pronounced gait abnormalities, including reduced homologous coordination during both the support and swing phases (Fig. 3B and C), whereas the braking index remained unchanged (Fig. 3D). In addition, DNP mice showed a decreased proportion of ground contact time within the step cycle (Fig. 3E), reduced average paw contact area (Fig. 3F), and increased instantaneous walking velocity (Fig. 3G), indicating impaired motor coordination and altered locomotor performance. PACAP-38 treatment markedly improved these gait abnormalities, as reflected by restored limb coordination, increased ground contact time and paw contact area, and normalization of walking velocity.

**Fig. 3:**
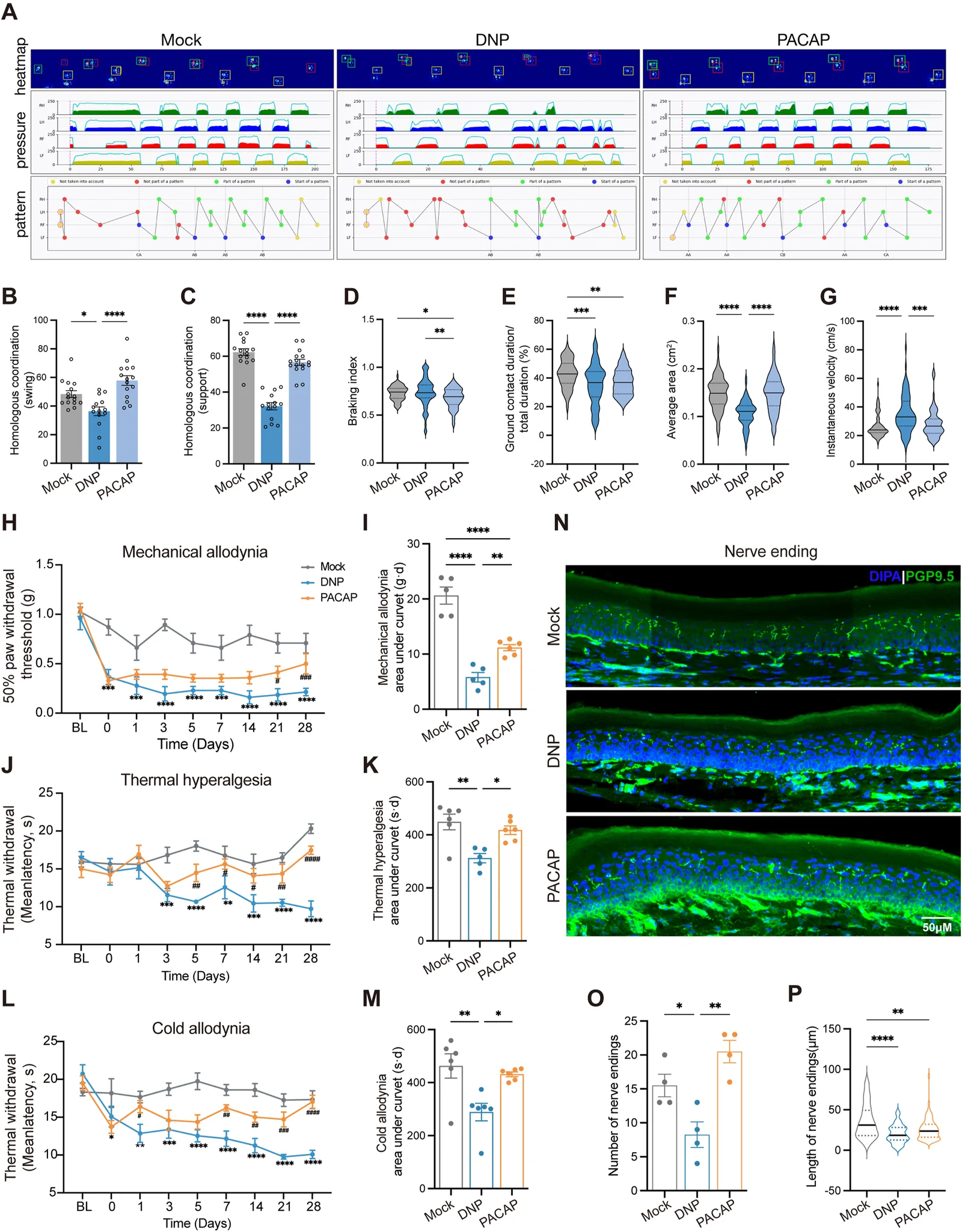
PACAP-38 improves gait dysfunction, neuropathic pain, and partially restores epidermal nerve fiber integrity in STZ-induced DNP mice.**(A)** Representative plantar pressure heatmaps and gait pattern traces from the Mock, DNP, and PACAP-38 groups, illustrating foot pressure distribution and locomotor patterns during walking. **(B**–**G)** Quantitative analysis of gait parameters: (B) homologous coordination (support phase); (C) homologous coordination (swing phase); (D) braking index; (E) ratio of ground contact duration to total step cycle; (F) average foot contact area; (G) instantaneous velocity. **(H, I)** Mechanical allodynia, assessed by the 50% paw withdrawal threshold, and corresponding AUC analysis. **(J, K)** Thermal hyperalgesia, assessed by thermal withdrawal latency, and AUC analysis. **(L, M)** Cold allodynia, assessed by cold withdrawal latency, and AUC analysis. **(N)** Representative immunofluorescence images of PGP9.5-positive epidermal nerve fibers in plantar skin (PGP9.5, green; DAPI, blue). Scale bar: 50 μm. **(O, P)** Quantification of (O) the number of epidermal nerve endings and (P) the average length of epidermal nerve fibers in plantar skin. Mean ± SEM. Statistical significance was assessed using one-way ANOVA followed by Tukey's post hoc test for gait parameters, AUC, and epidermal nerve fiber measurements, and two-way repeated-measures ANOVA for longitudinal behavioral measurements. ∗*p* < 0.05, ∗∗*p* < 0.01, ∗∗∗*p* < 0.001, ∗∗∗∗*p* < 0.0001.

Because nociceptive hypersensitivity is a key feature of DNP, we further assessed mechanical, thermal, and cold pain-related behaviors. DNP mice developed persistent mechanical allodynia, reflected by a progressive reduction in the 50% paw withdrawal threshold during the observation period (Fig. 3H). AUC analysis confirmed a significant increase in mechanical hypersensitivity in the DNP group (Fig. 3I). DNP mice also displayed thermal hyperalgesia and cold allodynia, as shown by shortened withdrawal latencies or enhanced responses to thermal and cold stimuli (Fig. 3J-M). PACAP-38 treatment significantly attenuated mechanical allodynia, thermal hyperalgesia, and cold allodynia, restoring paw withdrawal thresholds and latencies toward control levels.

To further examine the structural basis underlying these functional improvements, we assessed epidermal nerve fibers in plantar skin by PGP9.5 immunofluorescence staining (Fig. 3N). DNP mice showed a marked reduction in both the number and length of intraepidermal nerve fibers, indicating substantial peripheral denervation (Fig. 3O and P). PACAP-38-treated mice exhibited better preservation of epidermal nerve fibers, with increased nerve ending density and fiber length compared with untreated DNP mice. These results indicate that PACAP alleviates STZ-induced gait impairment, nociceptive hypersensitivity, and peripheral nerve degeneration.

### Transcriptomic profiling reveals metabolic and stress-related alterations associated with PACAP treatment in DNP

To define the molecular alterations associated with DNP and PACAP treatment, transcriptomic profiling was performed in DRG tissues. Overall gene expression distributions were comparable across samples, and PCA showed clear separation between the Mock and DNP groups (Fig. S2A and B). Differential expression analysis identified 389 DEGs between DNP and Mock mice and 161 DEGs between PACAP-treated and untreated DNP mice (FDR < 0.05, fold change >2; Fig. S2C and D). Venn analysis showed 91 overlapping DEGs, with 298 genes unique to DNP *vs*. Mock and 70 genes unique to PACAP *vs*. DNP comparisons (Fig. 4A), indicating distinct transcriptional responses associated with DNP and PACAP-38 treatment. KEGG enrichment analysis revealed that DNP-associated transcriptional changes were mainly related to neuronal signaling, calcium signaling, vascular-associated pathways, extracellular matrix interactions, and PI3K-Akt and cytokine-related signaling (Fig. 4B). In PACAP-treated DNP mice, differentially expressed genes were enriched in pathways involving extracellular matrix interactions, neuroactive ligand–receptor signaling, TGF-β, cytokine–cytokine receptor interactions, PI3K-Akt signaling, and cytoskeletal regulation (Fig. 4C). Additional GSEA and GO enrichment analyses are presented in Fig. S2E–L.

**Fig. 4:**
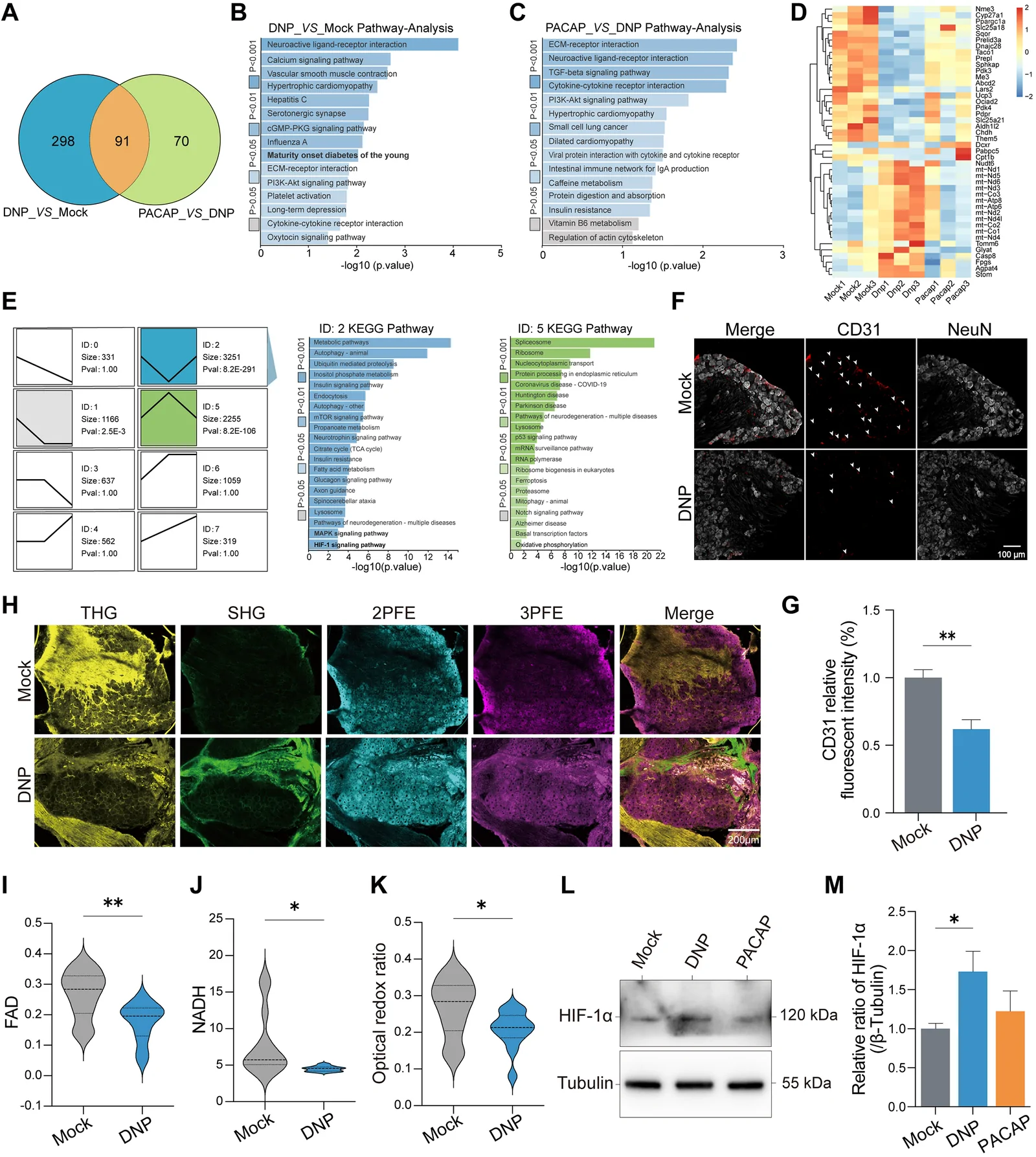
Transcriptomic and tissue-level analyses characterize molecular and metabolic alterations associated with DNP and PACAP-38 treatment.**(A)** Venn diagram showing the overlap of DEGs between the DNP vs. Mock and PACAP-38 *vs.* DNP comparisons. **(B, C)** KEGG pathway enrichment analysis of DEGs in the DNP *vs.* Mock (B) and PACAP *vs.* DNP (C) comparisons, showing enrichment of pathways related to neuroactive ligand–receptor interaction, calcium signaling, vascular smooth muscle contraction, cGMP-PKG signaling, PI3K-Akt signaling, and cytokine–cytokine receptor interaction. **(D)** Heatmap showing hierarchical clustering of mitochondria-associated DEGs identified using an FDR < 0.05 and an absolute fold-change cutoff of >1.5 across the Mock, DNP, and PACAP-38 groups. Mitochondrial-related genes were defined using Mouse MitoCarta 3.0 for re-analysis. Color intensity indicates normalized gene expression values. **(E)** Trend analysis of gene expression patterns across the Mock, DNP, and PACAP-38 groups, identifying two PACAP-associated reversed trends (ID2 and ID5), followed by KEGG pathway enrichment analyses of the genes within each trend. **(F)** Representative immunofluorescence images of CD31 and NeuN staining in DRG tissues from Mock and DNP mice. Arrowheads indicate CD31-positive endothelial cells. Scale bar = 100 μm. **(G)** Quantification of relative CD31 fluorescence intensity. **(H)** Representative femtosecond multiphoton label-free images of DRG tissues from Mock and DNP mice, including THG, SHG, 2PFE, 3PFE, and merged channels. Scale bar = 200 μm. **(I–K)** Quantification of label-free optical redox parameters, including NADH (I), FAD (J), and optical redox ratio [FAD/(NADH + FAD)] (K). **(L, M)** Representative Western blot images (L) and quantification (M) of HIF-1α protein expression in Mock, DNP, and PACAP-38 groups. Tubulin was used as the loading control. Data are presented as mean ± SEM. Statistical significance was assessed using an unpaired two-tailed Student's t-test for two-group comparisons and one-way ANOVA followed by Tukey's post hoc test for three-group comparisons. ∗*p* < 0.05, ∗∗*p* < 0.01.

To further investigate mitochondrial-related transcriptional changes, we performed a re-analysis of mitochondrial-related genes and carried out hierarchical clustering of all DEGs showing distinct expression patterns among Mock, DNP, and PACAP-38-treated samples (Fig. 4D). These data indicate widespread transcriptional perturbation of mitochondrial-component genes under DNP conditions. Trend analysis further identified PACAP-associated changes in metabolic and stress-related gene programs, including MAPK and HIF-1 signaling, insulin-related metabolism, oxidative phosphorylation, and neurodegeneration-related pathways (Fig. 4E). Together, these transcriptomic findings indicate broad neuronal, neurovascular, inflammatory, and metabolic transcriptional alterations in DNP, with PACAP-38 treatment associated with remodeling of these programs.

The transcriptomic findings were further examined at the tissue and metabolic levels. CD31 and NeuN co-staining showed a significant reduction in CD31-positive endothelial area in DRG from DNP mice, indicating impaired DRG vascular integrity (Fig. 4F and G). Femtosecond laser label-free imaging further confirmed metabolic redox alterations in DRG from DNP mice (Fig. 4H). Compared with Mock mice, DNP mice exhibited reduced NADH and FAD signals, together with a decreased redox ratio, suggesting a shift toward a reduced intracellular metabolic state (Fig. 4J–K). Western blot analysis demonstrated increased HIF-1α expression in DNP mice, which was partially attenuated by PACAP-38 treatment (Fig. 4L and M). Collectively, these findings indicate that DNP is associated with broad transcriptional alterations involving neuronal, vascular, metabolic, and stress-related pathways, accompanied by impaired DRG vascular integrity, altered metabolic redox status, and increased HIF-1α expression. PACAP-38 treatment was associated with partial normalization of these tissue and transcriptional alterations, supporting a potential role for PACAP in restoring the DRG microenvironment and metabolic homeostasis in DNP.

### PACAP-38 preserves mitochondrial homeostasis and modulates inflammatory responses in DRG neurons from DNP mice

We next examined whether PACAP protects DRG neurons by preserving mitochondrial homeostasis. Transmission electron microscopy revealed evident mitochondrial abnormalities in DRG neurons from DNP mice, including reduced mitochondrial numerical density, increased mitochondrial damage, and decreased mitochondrial area and perimeter compared with Mock mice (Fig. 5A–E). PACAP-38 treatment partially improved these ultrastructural alterations, particularly restoring mitochondrial area and perimeter, indicating preservation of mitochondrial morphology in DRG from DNP.

**Fig. 5:**
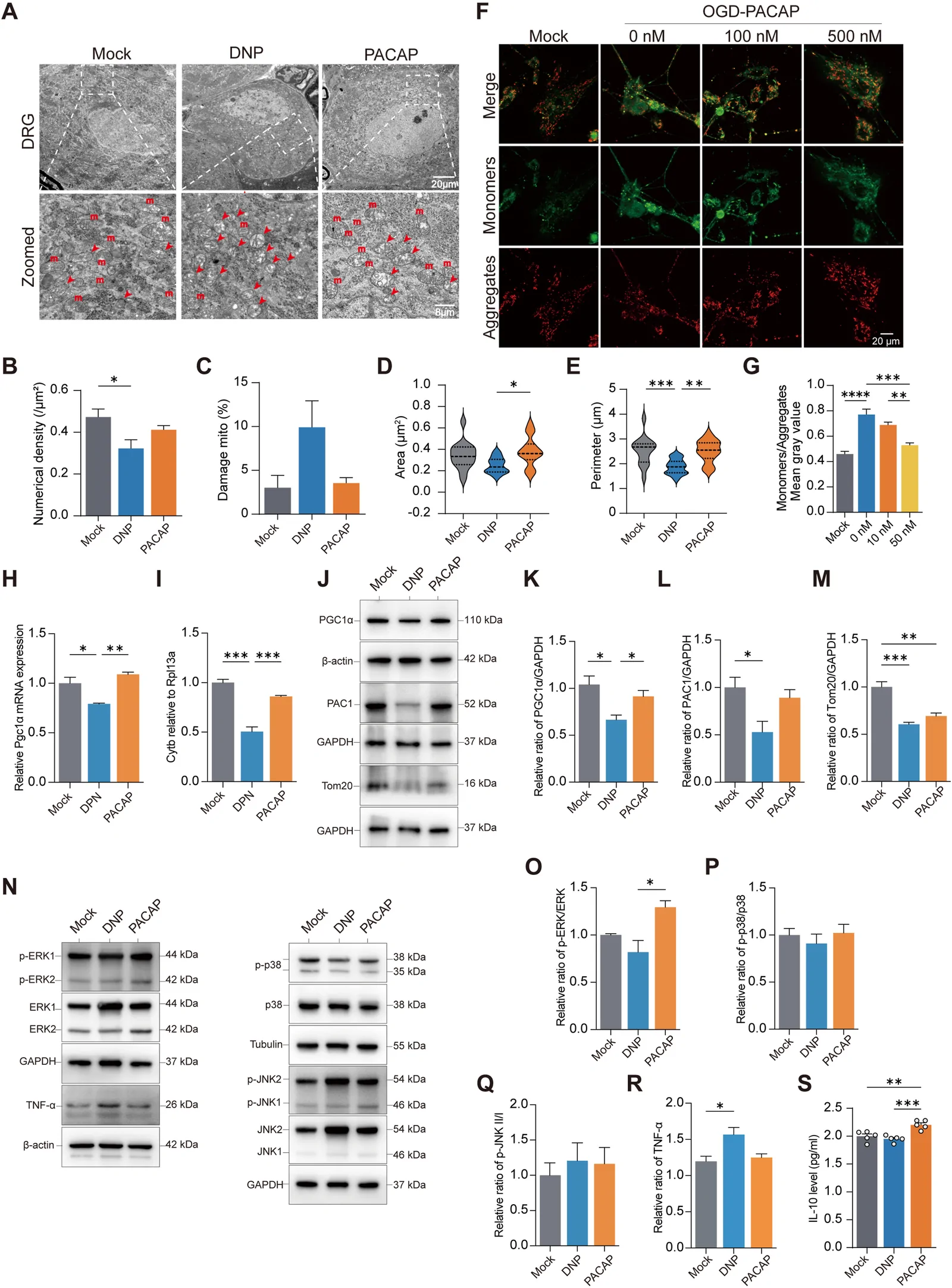
PACAP-38 improves mitochondrial integrity and modulates PGC1α-associated mitochondrial, ERK, and inflammatory responses in DNP mice.**(A)** Representative transmission electron microscopy images showing mitochondrial ultrastructure in DRG neurons from Mock, DNP, and PACAP-38-treated mice. Lower panels show enlarged views of mitochondrial morphology. Red arrowheads indicate damaged mitochondria, and “m” denotes morphologically intact mitochondria. Scale bars: 20 μm in the upper panels and 8 μm in the lower panels. **(B**–**E)** Quantitative analysis of mitochondrial density (B), mitochondrial damage rate (C), mitochondrial area (D), and mitochondrial perimeter (E). Mitochondrial density was defined as the number of mitochondria per μm^2^ of neuronal cross-sectional area. DNP mice exhibited reduced mitochondrial density, increased mitochondrial damage, and decreased mitochondrial size, whereas PACAP-38 treatment partially restored mitochondrial abnormalities. **(F)** Representative fluorescence images of mitochondrial membrane potential in DRG neurons exposed to OGD and treated with PACAP-38 at the indicated concentrations. Mitochondrial monomers and aggregates are shown in green and red, respectively. Scale bar: 20 μm. **(G)** Quantification of the mitochondrial monomer/aggregate ratio. OGD increased the monomer/aggregate ratio, indicating impaired mitochondrial membrane potential, whereas PACAP-38 treatment reduced this ratio. **(H, I)** RT-qPCR analysis of mitochondrial biogenesis-related genes, including *Pgc1α* (H) and mitochondrial DNA copy number (I), in DRG tissues. Mitochondrial copy number was presented as the ratio of mitochondrial DNA to nuclear DNA, where ΔCt = Ct _Cytochrome *b*_ − Ct _RPL13A_. PACAP-38 restored the DNP-induced downregulation of *Pgc1α* and *Cytb*. **(J)** Representative western blots showing the protein expression of PGC1α, PAC1 receptor, and Tom20 in DRG tissues from Mock, DNP, and PACAP-38-treated mice. β-actin or GAPDH was used as the loading control. **(K**–**M)** Quantitative analysis of PGC1α (K), PAC1 receptor (L), and Tom20 (M) protein expression. PACAP-38 increased PGC1α expression and partially restored PAC1 receptor and Tom20 levels in DNP mice. **(N)** Representative western blots showing phosphorylated and total ERK1/2, p38, and JNK, as well as TNF-α expression, in DRG tissues. GAPDH, β-actin, or tubulin was used as the loading control. **(O**–**Q)** Quantification of *p*-ERK/ERK (O), p-p38/p38 (P), and *p*-JNK/JNK (Q) ratios. PACAP significantly enhanced ERK phosphorylation, whereas p38 and JNK phosphorylation showed no significant changes among groups. **(R)** Quantitative analysis of TNF-α protein expression in DRG tissues. PACAP-38 treatment showed a reduction trend in TNF-α expression compared with the DNP group. **(S)** ELISA analysis of IL-10 levels in DRG tissues, showing that PACAP-38 increased IL-10 levels in DNP mice. Data are presented as mean ± SEM. ∗*p* < 0.05, ∗∗*p* < 0.01, ∗∗∗*p* < 0.001, ∗∗∗∗*p* < 0.0001.

To assess mitochondrial function under acute metabolic and hypoxic stress, mitochondrial membrane potential was evaluated in primary mouse DRG neurons exposed to OGD. OGD markedly increased the mitochondrial monomer/aggregate fluorescence ratio, indicating loss of mitochondrial membrane potential (Fig. 5F and G). PACAP-38 treatment reduced this increase in a concentration-dependent manner, suggesting that PACAP-38 alleviates OGD-induced mitochondrial dysfunction in DRG neurons from DNP mice. We further assessed mitochondrial biogenesis-related markers. RT-qPCR showed that *Pgc1α* and *Cytb* mRNA levels were significantly decreased in DNP mice and restored after PACAP-38 treatment (Fig. 5H and I). Consistently, Western blot analysis demonstrated reduced PGC1α protein expression in DNP DRGs, which was increased by PACAP-38 administration (Fig. 5J and K). PAC1 and Tom20 expression were also decreased in DNP mice and showed partial recovery following PACAP-38 treatment (Fig. 5J-M), indicating changes in PAC1 expression and mitochondrial content, respectively.

Given the involvement of MAPK signaling in neuronal stress responses and mitochondrial regulation, ERK, p38, and JNK activation were further examined. PACAP-38 significantly increased the *p*-ERK/ERK ratio compared with DNP mice, whereas p-p38/p38 and *p*-JNK/JNK ratios were not significantly altered among groups (Fig. 5N-Q). These results indicate selective activation of ERK signaling by PACAP-38 in the DRG from DNP. Finally, inflammatory mediators were assessed. TNF-α expression was increased in DNP mice compared with Mock controls, whereas PACAP-38 treatment showed a modest reduction trend, although this change did not reach statistical significance. (Fig. 5N and R). In contrast, IL-10 levels were significantly elevated after PACAP-38 administration (Fig. 5S), suggesting enhancement of anti-inflammatory signaling. Collectively, these findings indicate that PACAP ameliorates mitochondrial structural and functional abnormalities in the DRG of DNP mice, increases PGC1α-associated mitochondrial responses, enhances ERK phosphorylation, and modulates inflammatory mediators.

### PACAP-38 protects SH-SY5Y cells from OGD-induced oxidative stress and mitochondrial dysfunction

To further investigate the direct protective effects of PACAP against oxidative and mitochondrial stress, SH-SY5Y cells were subjected to OGD as an in vitro model of acute metabolic and hypoxic stress. We first optimized the duration of OGD exposure. Representative fluorescence imaging showed increased ROS-associated fluorescence with prolonged OGD exposure (Fig. S3A). JC-1 staining further revealed an increase in the monomer/aggregate fluorescence ratio following OGD, consistent with mitochondrial membrane depolarization (Fig. S3B and C). Quantitative analysis showed that the ratio was significantly increased after 3 and 5 h of OGD compared with Mock controls, with the most pronounced increase observed at 3 h. These findings confirmed that OGD induced robust oxidative and mitochondrial stress and identified 3 h as an appropriate duration for subsequent experiments.

We next characterized the transcriptional responses associated with OGD exposure. Transcriptomic profiling was performed after 1, 3, or 5 h of OGD (Fig. 6A). KEGG, GO, and GSEA analyses revealed broad alterations in neuronal, metabolic, and cellular stress-related programs at 3 h of OGD (Fig. 6B–D). Notably, the MAPK signaling pathway was enriched in the KEGG analysis, whereas GSEA identified diabetes-related gene signatures at 3 h of OGD (Fig. 6B–D). These findings indicate that OGD induces transcriptional changes involving signaling and metabolic processes that are relevant to, but do not directly recapitulate, the chronic pathological environment of DNP. Additional KEGG, GO, and GSEA analyses across the different OGD durations are presented in Fig. S4.

**Fig. 6:**
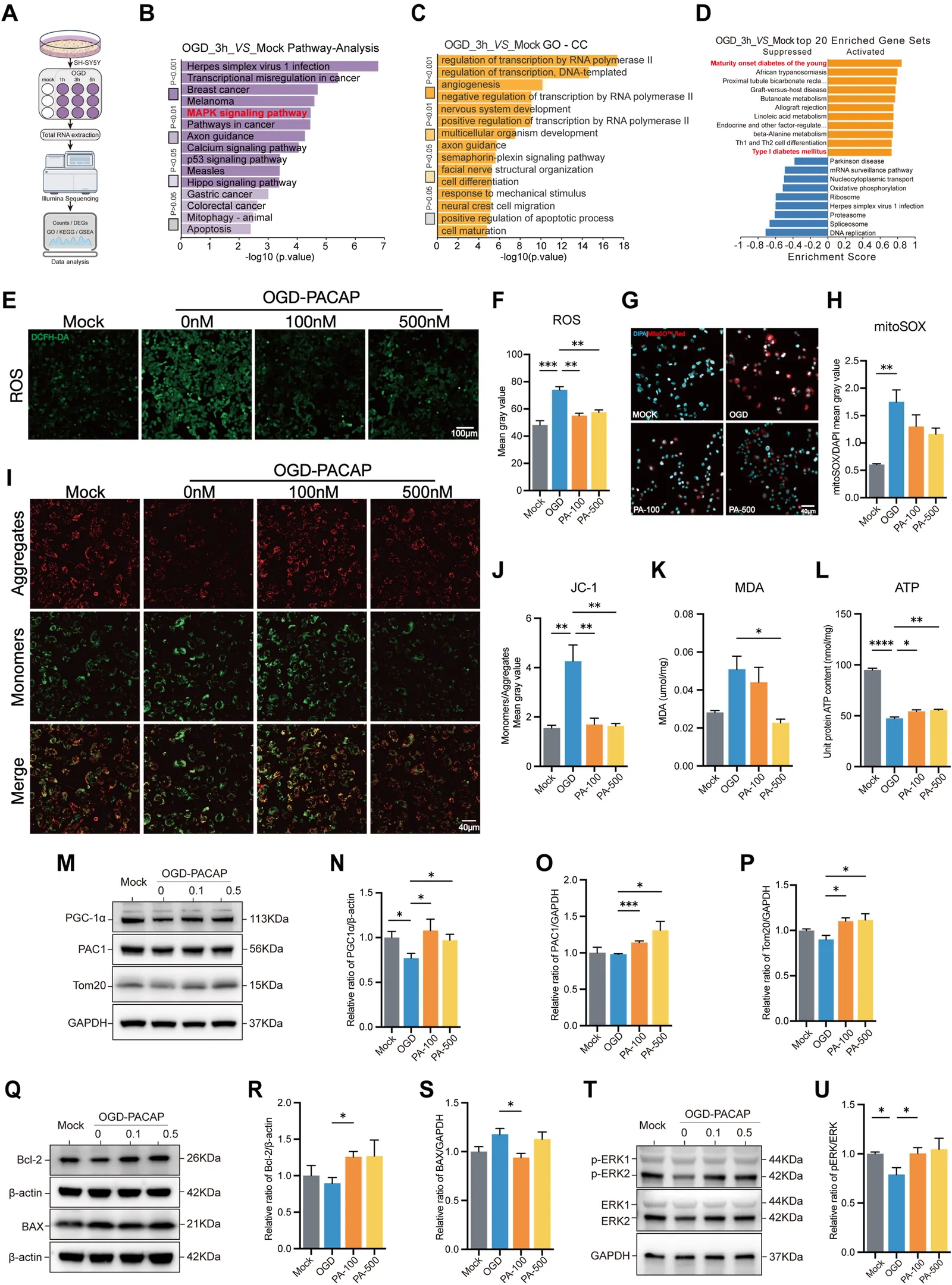
PACAP-38 alleviates OGD-triggered oxidative stress, mitochondrial impairment, and aberrant stress signaling in SH-SY5Y cells.**(A)** Schematic illustration of the in vitro experimental design. SH-SY5Y cells were subjected to OGD to induce metabolic and hypoxic stress, followed by PACAP-38 treatment at the indicated concentrations. **(B**–**D)** Transcriptomic analysis of SH-SY5Y cells exposed to 3 h of OGD compared with Mock controls. (B) KEGG pathway enrichment analysis of differentially expressed genes. (C) Gene Ontology cellular component (GO-CC) enrichment analysis. (D) Gene set enrichment analysis (GSEA) showing OGD-associated changes in selected biological programs. **(E)** Representative fluorescence images of intracellular ROS detected by DCFH-DA staining in Mock, OGD, OGD + PACAP-38 100 nM, and OGD + PACAP-38 500 nM groups. Scale bar: 50 μm. **(F)** Quantification of DCFH-DA fluorescence intensity showing intracellular ROS levels. **(G)** Representative fluorescence images of mitochondrial superoxide detected by mitoSOX Red staining. Nuclei were counterstained with DAPI. Scale bar: 40 μm. **(H)** Quantification of mitoSOX/DAPI fluorescence intensity, indicating mitochondrial superoxide anion production. **(I)** Representative JC-1 staining images showing mitochondrial membrane potential in SH-SY5Y cells. JC-1 aggregates are shown in red, and JC-1 monomers are shown in green. Scale bar: 40 μm. **(J)** Quantification of the JC-1 monomer/aggregate fluorescence ratio. **(K)** Quantification of malondialdehyde (MDA) levels. **(L)** Quantification of intracellular ATP content. **(M)** Representatives immunoblot bands of PGC1α, PAC1, Tom20, with GAPDH as internal loading control. **(N–P)** Densitometric quantification of PGC1α (N), PAC1 (O), and Tom20 (P) protein expression normalized to GAPDH. **(Q)** Western blot analysis of apoptotic regulatory proteins Bcl-2 and BAX; β-actin served as loading control. **(R**–**S)** Quantitative densitometry of Bcl-2 (R) and BAX (S) band intensities normalized to β-actin. **(T)** Representative immunoblots of total ERK1/2 and phosphorylated ERK1/2 (*p*-ERK1/2), with GAPDH as loading control. **(U)** Quantification of *p*-ERK1/2 normalized to total ERK1/2. Mean ± SEM. Statistical significance was determined by one-way ANOVA followed by Tukey's post-hoc test. ∗*p* < 0.05, ∗∗*p* < 0.01, ∗∗∗*p* < 0.001.

We then evaluated the concentration of PACAP-38 under the optimized 3 h OGD condition. CCK-8 analysis showed that PACAP-38 at 0.1 and 0.5 μM significantly increased cell viability compared with untreated OGD cells (Fig. S3D), and these concentrations were therefore used in subsequent experiments. PACAP-38 treatment also increased the mRNA expression of PAC1 and the neurotrophic factors MAGF and NG under OGD conditions (Fig. S3E–G), supporting a direct neuronal and neurotrophic response to PACAP-38.

Based on these findings, we examined whether PACAP-38 could attenuate OGD-induced oxidative stress and mitochondrial dysfunction in SH-SY5Y cells. OGD markedly increased intracellular ROS levels and mitochondrial superoxide production, as shown by DCFH-DA and MitoSOX staining, whereas PACAP-38 treatment reduced both signals (Fig. 6E–H). JC-1 staining further showed that OGD increased the mitochondrial monomer/aggregate ratio, indicating mitochondrial membrane potential depolarization; this effect was attenuated by PACAP-38 treatment (Fig. 6I and J). Consistently, OGD increased MDA levels and reduced ATP content, while PACAP decreased lipid peroxidation and partially restored cellular ATP levels (Fig. 6K and L).

We next examined mitochondrial regulatory responses associated with PACAP treatment. Western blot analysis showed that OGD reduced the expression of PGC1α, PAC1, and Tom20, suggesting impaired mitochondrial biogenesis and mitochondrial content. PACAP-38 treatment restored these mitochondrial-related proteins to varying degrees (Fig. 6M-P). These findings were consistent with the mitochondrial alterations observed *in vivo* and further supported the involvement of PGC1α-associated mitochondrial responses in PACAP-mediated protection. In addition, OGD disrupted apoptotic homeostasis by decreasing Bcl-2 and increasing BAX expression, whereas PACAP-38 partially reversed these changes (Fig. 6Q–S). PACAP-38 also restored OGD-suppressed ERK1/2 phosphorylation without markedly altering total ERK levels (Fig. 6T and U). Collectively, these results indicate that PACAP-38 protects SH-SY5Y cells against OGD-induced oxidative stress, mitochondrial membrane depolarization, energy failure, and apoptotic imbalance, and support a role for PGC1α-associated mitochondrial responses and ERK signaling in the cellular protective effects of PACAP-38.

### PACAP-38 alleviates neuropathic pain, with MEK/ERK inhibition attenuating its analgesic effects

To evaluate the *in vivo* therapeutic effects of PACAP-38 and explore whether ERK signaling contributes to its analgesic action, metabolic parameters and pain-related behaviors were assessed in Mock, DNP, PACAP-38-treated DNP, and PACAP-38 plus U0126-treated DNP mice (Fig. 7). U0126 was used as a pharmacological inhibitor of MEK1/2 to attenuate downstream ERK1/2 signaling. Compared with Mock mice, DNP mice showed reduced body weight gain during the 28-day observation period (Fig. 7A and B). PACAP-38 treatment did not significantly restore body weight gain, and co-administration of the ERK inhibitor U0126 had no additional effect. DNP mice also exhibited persistent hyperglycemia throughout the study period (Fig. 7C and D) and impaired glucose tolerance in the IPGTT performed on day 28 (Fig. 7E and F). Neither PACAP-38 alone nor PACAP-38 combined with U0126 markedly reduced blood glucose levels or improved glucose tolerance. These findings indicate that PACAP-38 does not substantially ameliorate systemic metabolic abnormalities in STZ-induced DNP mice.

**Fig. 7:**
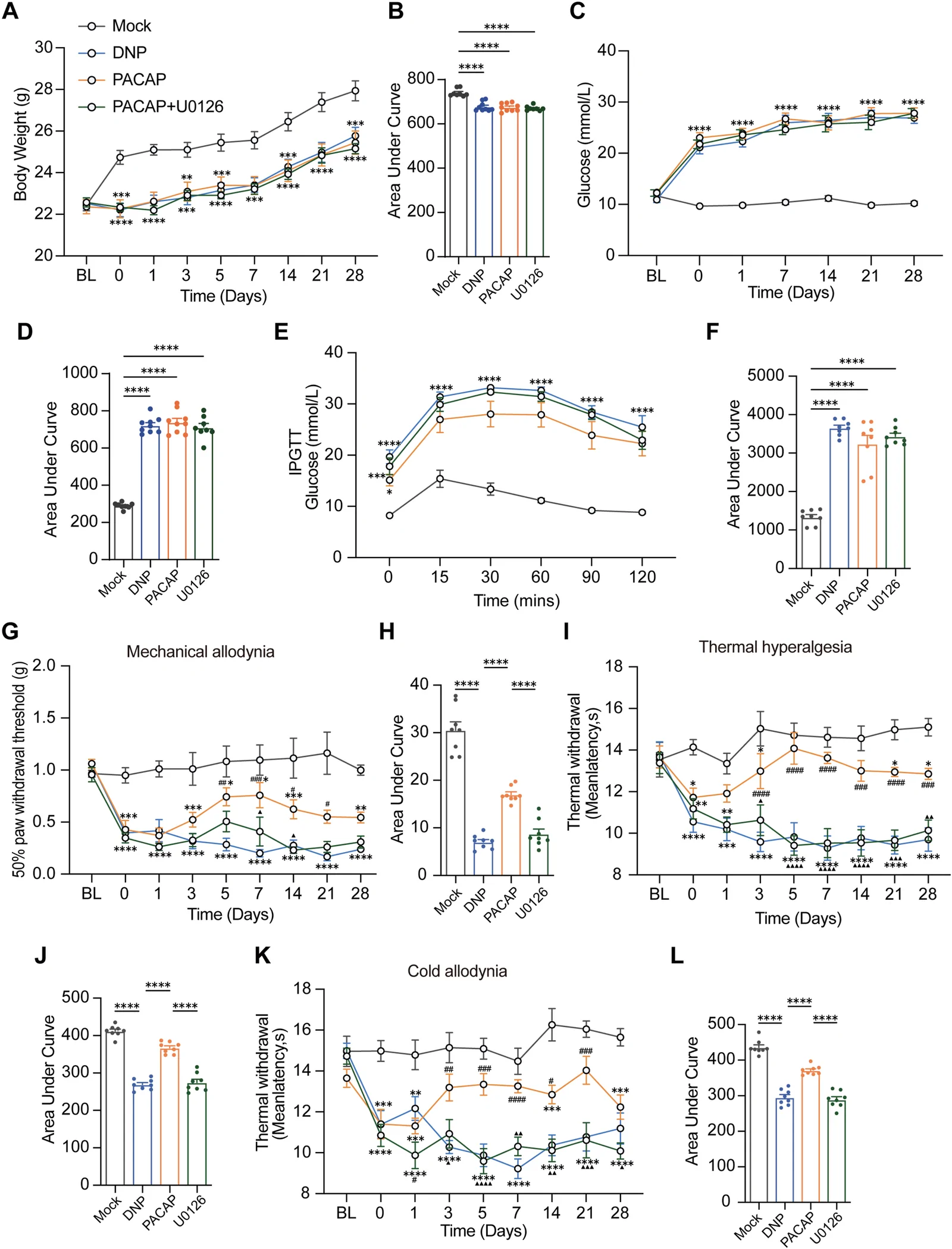
PACAP-38 alleviates DNP-associated neuropathic pain *in vivo*, with MEK/ERK inhibition attenuating its analgesic effects.**(A)** Body weight changes in Mock, DNP, PACAP-38, and PACAP-38 + U0126 (MEK1/2 inhibitor, 5 mg/kg) groups over the 28-day intervention period. **(B)** Quantification of the AUC for body weight changes in each group. **(C**–**D)** Blood glucose levels (C) and corresponding AUC (D) measured at baseline (BL) and indicated time points after treatment. **(E**–**F)** IPGTT results (E) and their AUC (F) at day 30, assessing systemic glucose homeostasis. **(G**–**H)** Mechanical allodynia measured by 50% paw withdrawal threshold (G) and its AUC (H). Asterisks indicate comparisons with Mock, hash symbols indicate comparisons with DNP, and triangles indicate comparisons with the PACAP-38 group. **(I**–**J)** Thermal hyperalgesia assessed by paw withdrawal latency (I) and its AUC (J). **(K**–**L)** Cold allodynia evaluated by paw withdrawal response (K) and its AUC (L). Data are presented as mean ± SEM. Statistical significance was determined as described in the Methods. ∗*p* < 0.05, ∗∗*p* < 0.01, ∗∗∗*p* < 0.001, ∗∗∗∗*p* < 0.0001.

In contrast to its limited effects on systemic metabolic parameters, PACAP-38 produced more pronounced improvements in pain-related behaviors. DNP mice developed persistent mechanical allodynia, as reflected by a sustained reduction in the 50% paw withdrawal threshold and a decreased area under the curve (Fig. 7G and H). PACAP-38 treatment significantly increased the paw withdrawal threshold, whereas U0126 co-treatment attenuated this effect, indicating that ERK inhibition reduced the analgesic action of PACAP. Similarly, DNP mice exhibited thermal hyperalgesia and cold allodynia, characterized by shortened withdrawal latencies to thermal and cold stimuli (Fig. 7I-L). PACAP-38 treatment alleviated both thermal and cold hypersensitivity, while U0126 partially reversed these protective effects. Collectively, these results indicate that PACAP-38 alleviates DNP-associated mechanical allodynia, thermal hyperalgesia, and cold allodynia without significantly improving body weight loss, hyperglycemia, or glucose intolerance. The attenuation of PACAP-38-associated analgesic effects by MEK/ERK inhibition suggests that ERK signaling contributes to the analgesic actions of PACAP-38 in DNP.

### ERK signaling contributes to PACAP-associated mitochondrial protection in DNP

Given the established involvement of ERK signaling in PACAP-associated cytoprotective responses, we next examined whether ERK signaling contributes to the mitochondrial protective effects of PACAP-38 in DNP. To this end, mitochondrial ultrastructure, membrane potential, mitochondrial biogenesis markers, and MAPK signaling were assessed following co-administration of PACAP-38 with U0126. Transmission electron microscopy showed that DRG neurons from DNP mice exhibited marked mitochondrial abnormalities, including reduced mitochondrial numerical density, increased mitochondrial damage, and decreased mitochondrial perimeter and area compared with Mock mice (Fig. 8A–E). PACAP-38 treatment partially improved mitochondrial morphology, whereas co-administration of U0126 attenuated these protective effects, indicating that ERK inhibition compromises PACAP-mediated preservation of mitochondrial ultrastructure.

**Fig. 8:**
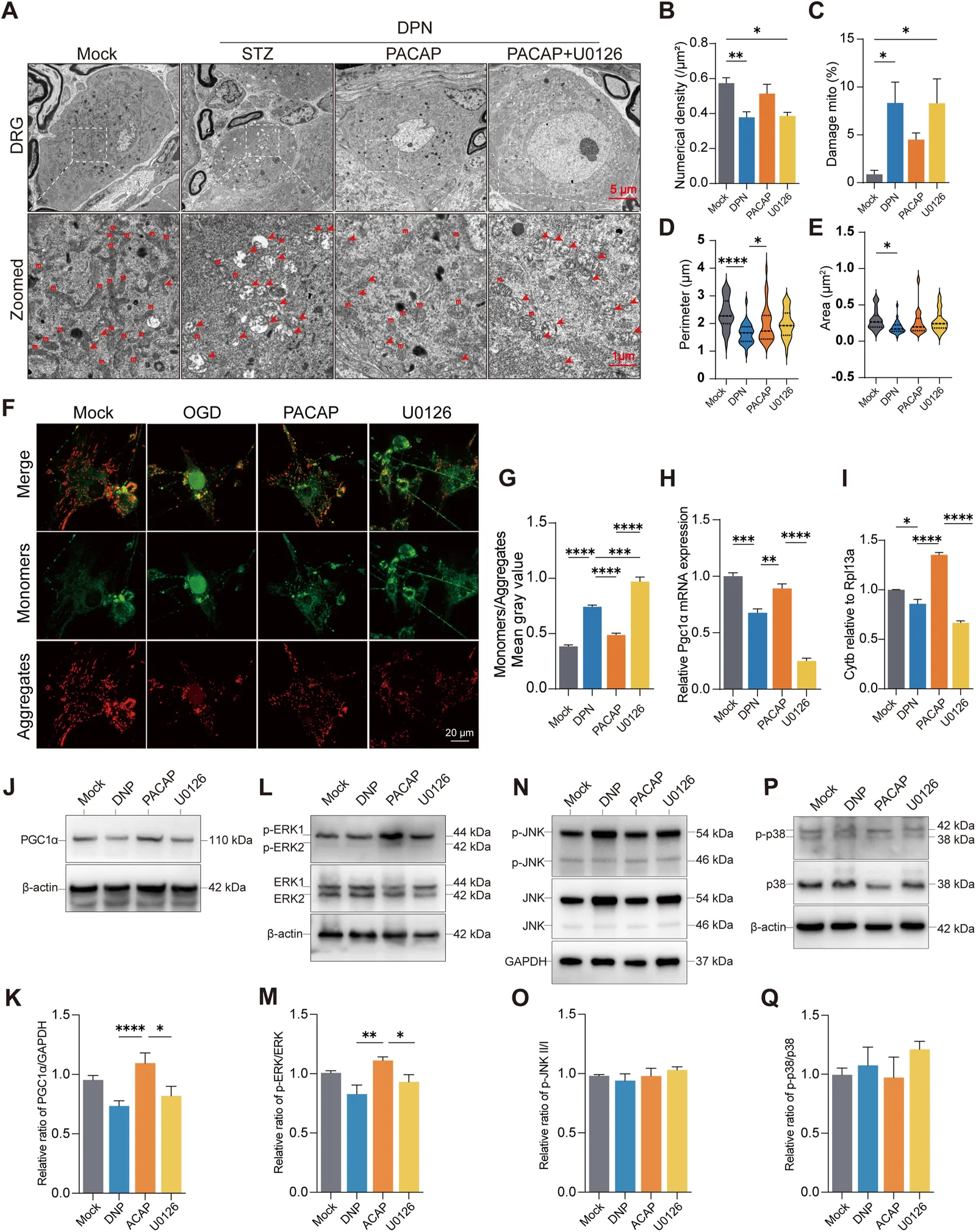
MEK/ERK inhibition attenuates PACAP-38-associated mitochondrial protection and PGC1α-related responses in DNP mice and OGD-exposed primary DRG neurons.**(A)** Representative transmission electron microscopy (TEM) micrographs of DRG neurons harvested from Mock, DNP, PACAP-38 -treated, and PACAP-38 + U0126 (ERK inhibitor) groups. Scale bars: 5 μm (upper overview images), 1 μm (high-magnification zoomed images); red arrowheads indicate damaged mitochondria, and “m” denotes morphologically intact mitochondria. **(B**–**E)** Quantitative statistics of mitochondrial morphological indexes based on TEM images: mitochondrial density (B), percentage of damaged mitochondria (C), mitochondrial perimeter (D), and mitochondrial cross-sectional area (E). **(F**–**G)** Confocal immunofluorescence staining and quantitative analysis of mitochondrial membrane potential in OGD-exposed cultured primary DRG neurons under Mock, OGD, PACAP-38, and PACAP-38 + U0126 treatments. Cells were stained with JC-1; scale bar = 20 μm. The monomer/aggregate fluorescence intensity ratio is shown in (G) **(H, I)** RT-qPCR quantification of relative mRNA levels of *Pgc1α* (H) and *Cytb* (normalized to *Rpl13a*) in mouse DRG tissues across four experimental groups. **(J**–**Q)** Western blot detection and densitometric quantification of PGC1α, total/phosphorylated ERK1/2, total/phosphorylated JNK, and total/phosphorylated p38 in DRG lysates. Β-actin or GAPDH was applied as internal loading control for protein normalization (K/M/O/Q for quantitative statistics). Data are presented as mean ± SEM. Statistical significance was determined by one-way ANOVA followed by Tukey's post hoc test. ∗*p* < 0.05, ∗∗*p* < 0.01, ∗∗∗*p* < 0.001, ∗∗∗∗*p* < 0.0001.

Consistently, JC-1 staining in the OGD model showed that OGD increased the mitochondrial monomer/aggregate ratio, reflecting mitochondrial membrane potential depolarization (Fig. 8F and G). PACAP-38 reduced this ratio, whereas co-treatment with U0126 attenuated the protective effect of PACAP-38. Mitochondrial biogenesis-related markers were then examined. DNP decreased *Pgc1α* mRNA expression, while PACAP-38 restored *Pgc1α* levels and increased *Cytb* expression (Fig. 8H and I). These effects were suppressed by U0126. Western blot analysis further confirmed that PGC1α protein expression was reduced in DNP mice, restored by PACAP-38, and decreased again after ERK inhibition (Fig. 8J and K). These findings indicate that ERK inhibition attenuates PACAP-associated PGC1α responses, supporting a potential relationship between ERK signaling and PGC1α-associated mitochondrial regulation.

Finally, Western blot analysis showed that PACAP-38 increased the *p*-ERK/ERK ratio, whereas U0126 blocked PACAP-induced ERK activation (Fig. 8L and M). In contrast, phosphorylation of JNK and p38 was not significantly altered among groups (Fig. 8N-Q), suggesting selective involvement of ERK signaling. Collectively, these results demonstrate that pharmacological inhibition of MEK/ERK signaling attenuated several of these PACAP-associated effects, supporting a contribution of MEK/ERK signaling to PACAP-mediated mitochondrial protection and PGC1α-associated responses.

### ERK signaling contributes to PACAP-mediated mitochondrial protection *in vitro*

To determine whether ERK1/2 signaling contributes to PACAP-mediated mitochondrial protection at the cellular level, SH-SY5Y cells were subjected to OGD and treated with PACAP-38 in the presence or absence of the ERK inhibitor U0126 (Fig. 9). OGD markedly increased intracellular ROS levels, whereas PACAP treatment significantly reduced ROS accumulation (Fig. 9A and B). This antioxidant effect was attenuated by U0126, indicating that ERK inhibition compromises PACAP-mediated suppression of oxidative stress. Consistently, OGD increased MDA levels, which were reduced by PACAP but increased again after U0126 co-treatment (Fig. 9C). Mitochondrial function was assessed by JC-1 staining and ATP measurement. OGD increased the JC-1 monomer/aggregate ratio, indicating mitochondrial membrane potential depolarization (Fig. 9D and E). PACAP significantly lowered this ratio, whereas U0126 reversed this protective effect. In parallel, OGD reduced cellular ATP content, and PACAP partially restored ATP levels; this effect was also attenuated by U0126 (Fig. 9F). These findings suggest that ERK signaling contributes to the maintenance of mitochondrial membrane potential and cellular energy metabolism during PACAP-38 treatment under OGD stress.

**Fig. 9:**
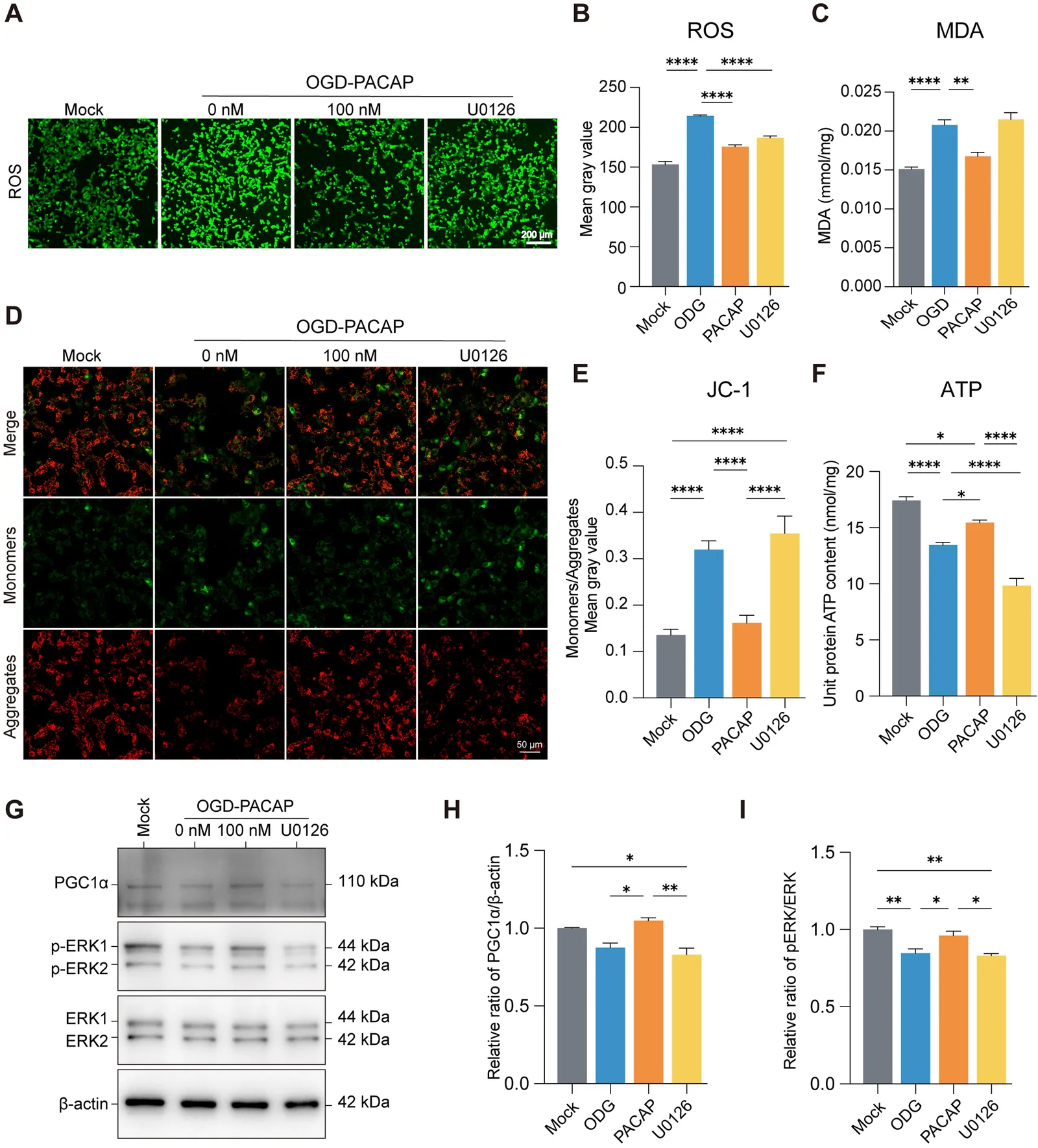
MEK/ERK inhibition attenuates PACAP-38-associated protection against OGD-induced oxidative stress and mitochondrial dysfunction in SH-SY5Y cells.**(A)** Representative DCFH-DA fluorescence images showing intracellular ROS levels in Mock, OGD, OGD + 100 nM PACAP-38, and OGD + 100 nM PACAP-38 + 10 μM U0126 groups. Scale bar = 200 μm. **(B)** Quantification of DCFH-DA fluorescence intensity. **(C)** Quantification of malondialdehyde (MDA) levels. **(D)** Representative JC-1 fluorescence images showing mitochondrial membrane potential. Red fluorescence indicates JC-1 aggregates, whereas green fluorescence indicates JC-1 monomers. Scale bar = 50 μm. **(E)** Quantification of the JC-1 monomer/aggregate fluorescence ratio. **(F)** Quantification of cellular ATP content. **(G)** Representative Western blot images of PGC1α, phosphorylated ERK1/2, total ERK1/2, and β-actin in each group. **(H)** Quantification of relative PGC1α protein expression. **(I)** Quantification of the *p*-ERK1/2/ERK1/2 ratio. All quantitative data are presented as mean ± SEM. Statistical comparisons were performed using one-way ANOVA followed by Tukey's multiple comparison post-hoc test. ∗*p* < 0.05, ∗∗*p* < 0.01, ∗∗∗∗*p* < 0.0001.

Western blot analysis further showed that OGD reduced PGC1α expression and ERK phosphorylation (Fig. 9G–I). PACAP restored both PGC1α protein levels and the *p*-ERK/ERK ratio, whereas U0126 suppressed PACAP-induced ERK activation and reduced PGC1α expression. Collectively, these findings indicate that PACAP-38 attenuates OGD-induced oxidative stress, mitochondrial membrane depolarization, and ATP depletion, and that these protective effects are partially attenuated by pharmacological ERK inhibition. The concomitant changes in ERK phosphorylation and PGC1α expression further support a contribution of ERK signaling to PACAP-associated PGC1α responses and mitochondrial protection in SH-SY5Y cells.

## Discussion

DNP remains a clinically challenging complication of diabetes because current treatments mainly provide symptomatic analgesia and do not sufficiently target the mechanisms that drive nerve degeneration. Accumulating evidence indicates that DPN is not simply a consequence of chronic hyperglycemia, but a complex neurovascular and metabolic disorder involving mitochondrial dysfunction, oxidative stress, impaired axonal energy supply, inflammatory activation, endothelial injury, and progressive degeneration of sensory neurons and their peripheral terminals [2], [6], [29], [30]. In the present study, we combined clinical observations with *in vivo*, transcriptomic, ultrastructural, and complementary cellular experiments to investigate the potential protective effects of PACAP-38 in DNP. Our findings support a model in which DNP is characterized by systemic inflammatory activation, reduced circulating PACAP-38 levels, DRG endothelial impairment, hypoxia-related metabolic stress, redox imbalance, mitochondrial injury, and peripheral nerve fiber loss. PACAP-38 treatment alleviated neuropathic pain and improved peripheral nerve and mitochondrial phenotypes. Mechanistically, PACAP-38 increased ERK phosphorylation and PGC1α expression, whereas ERK inhibition attenuated several PACAP-associated effects. These findings suggest that ERK signaling may contribute to PACAP-associated regulation of PGC1α and mitochondrial homeostasis in DNP, while leaving open the involvement of additional signaling pathways and PACAP receptor subtypes.

A notable clinical observation was the altered circulating PACAP-38 profile across the clinical groups. Serum PACAP-38 levels were significantly higher in patients with T2DM than in healthy controls and patients with DNP. DNP patients showed numerically higher PACAP-38 levels versus healthy controls, without statistical significance. These findings differ from those reported by Yevgi et al. who observed higher serum PACAP levels in patients with painful polyneuropathy, including both diabetic and non-diabetic cases, than in healthy controls [31]. However, that study did not include a group with uncomplicated T2DM, limiting direct comparison. The elevation of PACAP-38 in T2DM without neuropathy may reflect an adaptive response to metabolic stress, consistent with the reported roles of PACAP in neuroendocrine regulation, islet function, and cellular stress resistance [14], [32], [33]. Nevertheless, current clinical evidence does not establish a consistent pattern of circulating PACAP changes in neuropathy, and differences in disease context, patient characteristics, and study design may contribute to the variation across studies. In our cohort, altered circulating PACAP-38 levels coexisted with elevated pro-inflammatory cytokines, including IFN-γ, IL-17A, TNF-α, and IL-6, indicates that changes in circulating PACAP-38 levels accompany inflammatory activation in DNP, but does not establish a causal relationship between them. However, the cross-sectional design of our study does not allow us to determine whether altered PACAP-38 level is a cause, consequence, or biomarker of neuropathy progression, or whether these alterations are causally related to inflammatory activation. Larger prospective studies are needed to clarify whether circulating PACAP-38 can predict DNP onset, progression, or severity.

In the present study, PACAP-38 had limited effects on systemic metabolic abnormalities. Although a modest improvement in body-weight trajectory was observed in the primary therapeutic cohort, this effect was not consistently reproduced in the separate mechanistic cohort. Moreover, PACAP-38 did not significantly improve persistent hyperglycemia or impaired glucose tolerance, nor did it ameliorate pancreatic islet damage. This findings are consistent with previous evidence that PACAP has limited effects on systemic glucose regulation, including observations in STZ-diabetic rodents [22] and evidence that PACAP plays a limited role in basal glucose homeostasis [34]. However, low-dose PACAP-38 has been reported to prevent the development of hyperglycemia in GK rats [35], suggesting that its metabolic effects may depend on the disease model, dose, administration regimen, and timing of intervention.

Despite its limited effects on systemic metabolic parameters, PACAP has previously been shown to exert neurotrophic and neuroprotective effects. In rat models of diabetic neuropathy, PACAP-38 has been reported to ameliorate neuropathic phenotypes, reduce mechanical hyperalgesia, and inhibit mitochondrial fission [22]. Repeated intrathecal administration of PACAP-38 relieves pain and promotes axonal regeneration [36]. In addition, our previous study in a paclitaxel-induced peripheral neuropathy model showed that PACAP alleviated neuropathic pain and improved mitochondrial function [25]. Consistent with these findings, PACAP-38 improved gait abnormalities and reduced mechanical allodynia, thermal hyperalgesia, and cold allodynia in our DNP model. These behavioral improvements were accompanied by preservation of PGP9.5-positive intraepidermal nerve fibers in plantar skin, suggesting that PACAP not only reduces pain hypersensitivity but also protects peripheral nerve integrity. Because reduced intraepidermal nerve fiber density is a recognized structural correlate of small-fiber neuropathy and is widely used in the evaluation of painful neuropathies [37], [38], the preservation of epidermal innervation provides morphological support for the functional improvements induced by PACAP. Taken together, these findings support the neuroprotective effects of PACAP-38 in DNP, which appear to be largely independent of improvements in systemic glucose metabolism.

Transcriptomic profiling provided additional insight into the pathological state of DRGs in DNP. DNP-associated transcriptional changes were enriched in pathways related to neuroactive ligand-receptor interaction, calcium signaling, vascular smooth muscle contraction, extracellular matrix organization, oxidative phosphorylation, and metabolic regulation. Consistently, CD31 and NeuN co-staining showed reduced CD31-positive endothelial area in DRGs from DNP mice, indicating local vascular impairment. In parallel, increased HIF-1α expression, reduced NADH and FAD signals, and a decreased optical redox ratio further suggest hypoxia-associated metabolic stress and disrupted redox homeostasis. These findings support the presence of vascular-associated and metabolic-redox abnormalities in the DRG microenvironment in DNP. Transcriptomic analyses further revealed PACAP-associated changes in gene-expression patterns related to metabolic regulation and cellular stress responses, including pathways related to metabolic regulation, fatty acid metabolism, MAPK and HIF-1 signaling, as well as oxidative phosphorylation and ferroptosis. Hyperglycemia-induced mitochondrial overload, impaired oxidative phosphorylation, and redox imbalance have been recognized as major mechanisms contributing to diabetic neuropathy [6], [29], [30]. Our findings extend this concept by showing that DNP involves not only mitochondrial injury in sensory neurons, but also broader disruption of the DRG neurovascular and metabolic microenvironment. These findings suggest that PACAP-associated transcriptional changes provide complementary molecular evidence of altered metabolic and stress-related processes in diabetic DRGs.

Mitochondrial dysfunction appears to be a central mechanism linking diabetes to DRG neuronal injury and pain hypersensitivity. Sensory neurons have long axons and high energetic demands, making them particularly vulnerable to mitochondrial damage, impaired axonal transport, and ATP deficiency [6], [30], [39]. Recent studies have demonstrated that the neuroprotective effects of PACAP are tightly linked to mitochondrial homeostasis [40]. Previous studies have shown that PACAP protects against mitochondrial dysfunction in experimental neuropathy, including attenuation of mitochondrial fission and mitochondrial damage [22], [25]. Moreover, PACAP-induced mitochondrial activation has been linked to PGC1α, with PGC1α knockdown attenuating PACAP-induced mitochondrial activation and neurite outgrowth [23]. In this study, TEM revealed reduced mitochondrial numerical density, increased mitochondrial damage, and decreased mitochondrial area and perimeter in DRG neurons from DNP mice. These structural abnormalities were accompanied by reduced expression of mitochondrial biogenesis-related markers, including PGC1α and downstream mitochondrial genes, as well as decreased Tom20 expression. PGC1α is a master regulator of mitochondrial biogenesis and oxidative metabolism, and impaired PGC1α-related signaling has been implicated in metabolic and neurodegenerative disorders [41], [42]. Together, these findings support a protective role for PACAP-38 in mitochondrial homeostasis under diabetic and metabolic stress conditions and suggest that PGC1α-associated regulation may contribute to these effects.

Previous studies have shown that PACAP can activate ERK signaling [16], [17], and ERK signaling has been implicated in PACAP-mediated neuroprotection and mitochondrial protection [21]. PACAP-induced ERK activation can involve distinct mechanisms, including cAMP-dependent Rapgef2/Rap1/B-Raf signaling [17] and PAC1 receptor internalization and PLC/PKC-dependent signaling [43]. In the present study, PACAP-38 increased ERK phosphorylation in the DRG without significantly affecting p38 or JNK. Pharmacological inhibition of MEK/ERK signaling with U0126 attenuated the PACAP-38-associated improvements in pain behaviors, mitochondrial ultrastructure, PGC1α and Cytb expression, and mitochondrial membrane potential. These findings support the involvement of ERK signaling in PACAP-mediated mitochondrial protection and PGC1α-associated responses in DNP. However, PACAP receptors engage multiple downstream signaling pathways, including cAMP/PKA, PLC, and MAPK/ERK pathways [14], [44]. Therefore, the present findings do not exclude contributions from other PACAP-responsive pathways, particularly cAMP/PKA signaling, which may also contribute to PGC1α regulation. Further studies are required to determine the relationship between MEK/ERK and other PACAP-responsive pathways and to clarify the downstream mechanisms linking PACAP/ERK signaling to PGC1α.

Recent studies have demonstrated that PACAP suppresses inflammatory responses, alleviates oxidative stress and reduces cellular apoptosis, with several reports linking these protective effects to the preservation of mitochondrial homeostasis [45], [46], [47]. Another important aspect of this study is the relationship between mitochondrial protection and inflammation. In DNP mice, TNF-α expression was increased, consistent with enhanced pro-inflammatory signaling. PACAP-38 treatment was associated with a modest downward trend in TNF-α expression and significantly increased IL-10 levels, suggesting a potential role for PACAP in modulating the inflammatory milieu in the DNP model. Mitochondrial dysfunction can amplify inflammation through increased mitochondrial danger signals, altered redox state, and activation of inflammatory transcriptional programs [6], [29], while inflammatory cytokines may further compromise mitochondrial function and influence sensory neuron excitability. The concurrent changes in mitochondrial and inflammatory measures observed in our study raise the possibility that PACAP-38 may influence the interplay between these processes during DNP. Therefore, PACAP may interrupt a feed-forward cycle in which mitochondrial injury and inflammation reinforce each other during DNP. This interpretation is consistent with previous reports that PACAP exerts anti-inflammatory and cytoprotective effects in neural and peripheral tissues [14], [48]. However, whether the anti-inflammatory effects of PACAP-38 are directly mediated by mitochondrial protection, or whether these processes are regulated in parallel, remains to be determined.

From a translational perspective, PACAP may represent a potential therapeutic candidate for DNP because it modulates multiple pathological processes, including mitochondrial dysfunction, oxidative stress, inflammation, vascular impairment, and neuronal injury. However, despite growing interest in PACAP as a neuroprotective agent, its therapeutic development for neurological and neurodegenerative disorders remains largely at the preclinical stage [49]. Future therapeutic development should therefore benefit from receptor-selective agonists, more stable PACAP analogues, sustained-release formulations, or targeted delivery strategies to improve efficacy and reduce systemic side effects. Several limitations should also be noted. The clinical sample size was relatively small and cross-sectional, precluding conclusions regarding the causal or temporal relationship between circulating PACAP-38 levels and DNP. The STZ-induced model does not fully recapitulate the metabolic complexity of type 2 diabetic neuropathy, particularly obesity, insulin resistance, and dyslipidemia [34,35]. In addition, this study focused mainly on DRG neurons, whereas diabetic neuropathy also involves Schwann cells, immune cells, endothelial cells, peripheral axons, and spinal circuits. Although U0126 attenuated several PACAP-associated protective effects, the absence of a U0126-alone group and the potential effects of ERK inhibition on nociceptive signaling prevent us from assigning these effects exclusively to blockade of PACAP signaling. The downstream mechanism linking ERK activation to PGC1α induction remains to be further defined.

In conclusion, this study shows that DNP is associated with systemic inflammation, altered circulating PACAP-38 levels, endothelial impairment, hypoxia-related stress, redox imbalance, mitochondrial injury, and peripheral nerve fiber loss. PACAP-38 alleviates neuropathic pain, accompanied by improved mitochondrial homeostasis and activation of ERK signaling, without substantially correcting systemic hyperglycemia. Pharmacological inhibition of MEK/ERK attenuated several PACAP-associated protective effects, supporting a contribution of ERK signaling to PACAP-mediated mitochondrial protection and PGC1α-associated responses. These findings highlight the potential of PACAP-38 as a candidate for further investigation in diabetic neuropathy and warrant additional studies to clarify its underlying mechanisms and therapeutic relevance.

## Author contributions

The authors’ contributions were as follows: W Sun, M Zhai and L Xiao were responsible for the conceptualization and design of the study, data analysis, interpretation, and manuscript writing. D Cao, Q Zhang, S Shi, L Jiang, B Gong, S Yang, Z Luo and S Guo conducted animal experiments; W Sun, M Zhai, L Xiao, D Cao, and Q Zhang reviewed the manuscript. W Sun, M Zhai, D Cao and L Xiao obtained financial support from grants.

## Ethics approval and consent to participate

The human study protocol was reviewed and approved by the Ethics Committee of the Fifth Affiliated Hospital, Southern Medical University (approval No. 2025-JYYXK-J-002). The study was conducted in accordance with the Declaration of Helsinki, and written informed consent was obtained from all participants. All animal experiments were reviewed and approved by the Institutional Animal Care and Use Committee (IACUC) of Nanshan People's Hospital, Shenzhen (approval No. NSHP-JY-25015), and were performed in accordance with institutional guidelines for the care and use of laboratory animals.

## Consent for publication

The paper has been read and approved by all authors. All authors approved the submission of this paper for publication. All authors confirmed that neither the manuscript submitted nor any part of it has been published or is being considered for publication elsewhere.

## Funding

This work was supported by grants from the National Natural Science Foundation of China (No. 82171378, No.82401438), Science, Technology and Innovation Commission of Shenzhen Municipality (No. JCYJ20240813114512016, No. JCYJ20240813152049062; No. JCYJ20240813114612017; JCYJ20240813114552067), Shenzhen Nanshan District Healthcare System Science and Technology Key Projects (No. NSZD2023003; No. NSZD2024007; No. NSZD2025007; No. NS2025008).

## Declaration of competing interest

The authors declare that they have no known competing financial interests or personal relationships that could have appeared to influence the work reported in this paper.

## Figures and Tables

**Figure:**
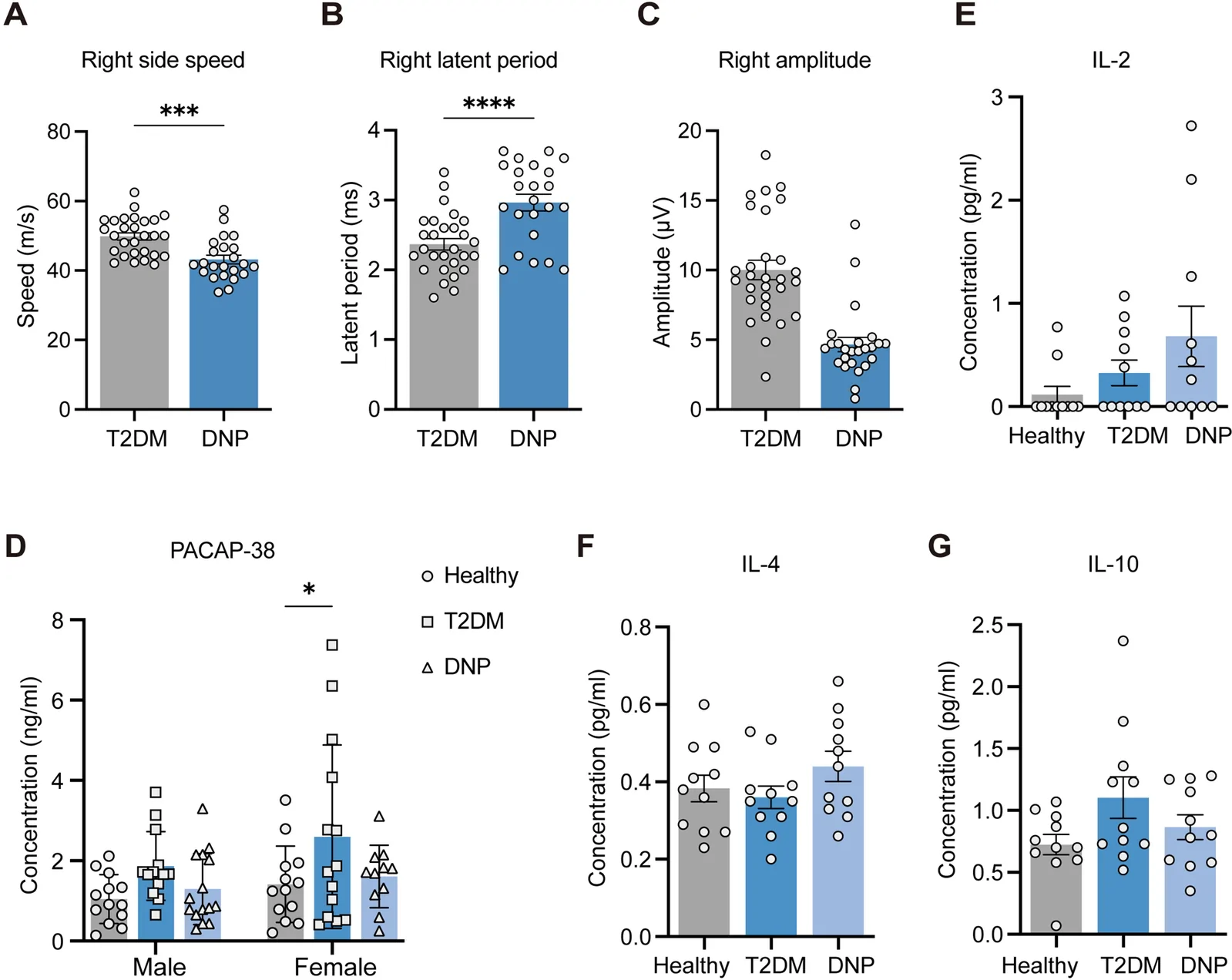
Fig. S1**Supplementary analyses of serum inflammatory cytokines and circulating PACAP-38 levels across clinical cohorts.** **(A**–**C)** Quantitative analysis of neurophysiological parameters: (A) sensory nerve conduction velocity, (B) distal latency, and (C) SNAP amplitude for the right sural nerves in T2DM and DNP patients. **(D)** Two-way ANOVA of serum PACAP-38 concentrations with clinical group and sex as factors. A significant main effect of clinical group was observed (P = 0.0098), while neither the main effect of sex (P = 0.0917) nor the clinical group ⨉ sex interaction reached statistical significance. **(E**–**G)** Serum levels of (E) IL-2, (F) IL-4, and (G) IL-10 in healthy controls, patients with type 2 diabetes mellitus (T2DM), and patients with diabetic neuropathic pain (DNP). Data are presented as mean ± SEM.

**Figure:**
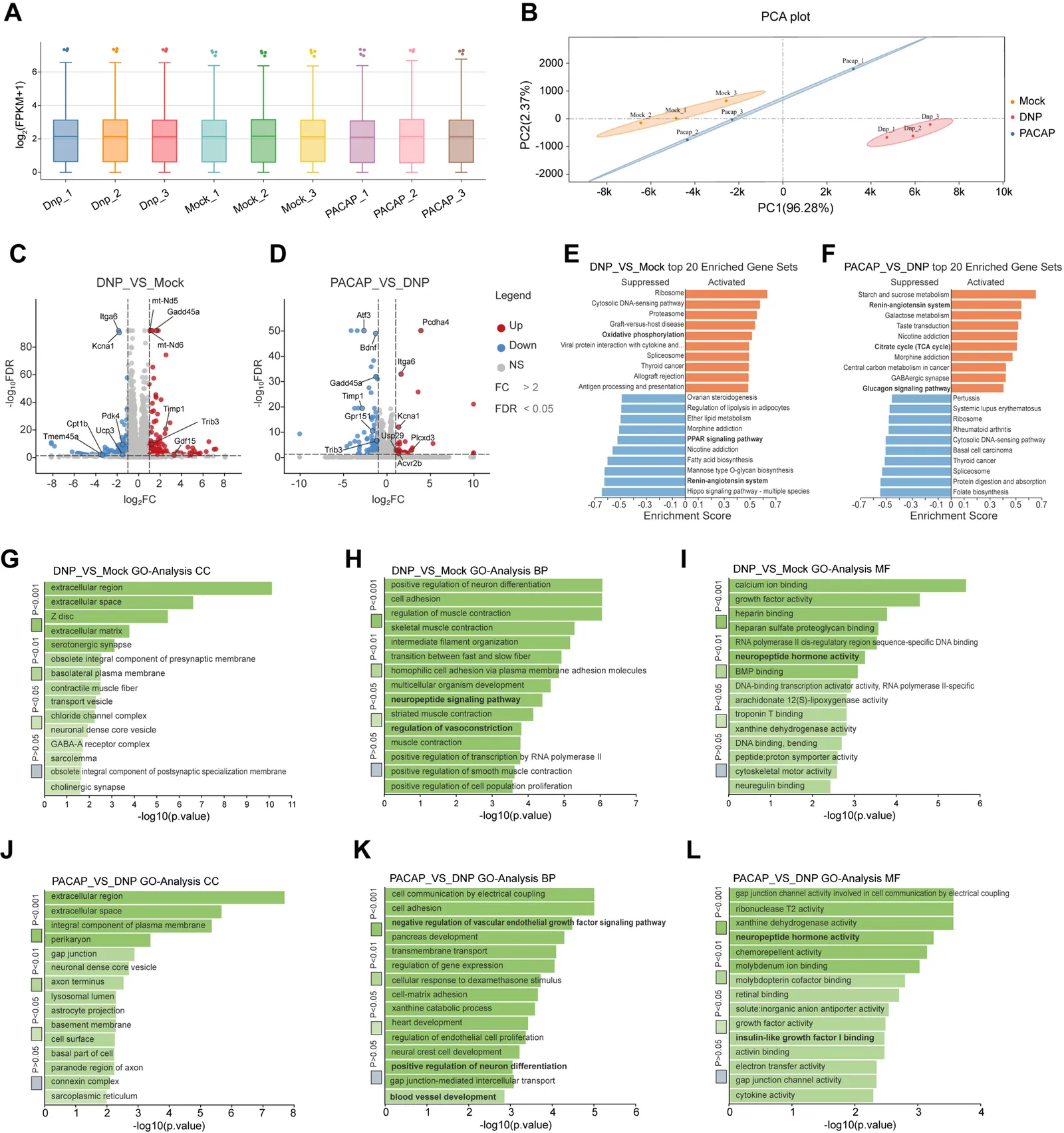
Fig. S2**Quality assessment and functional enrichment analysis of DRG transcriptomic profiles.** **(A)** Distribution of gene expression levels across individual DRG samples, shown as log_2_ (FPKM + 1). **(B)** Principal component analysis (PCA) of DRG transcriptomic profiles. The Mock and DNP samples showed clear separation, whereas the PACAP-38-treated samples displayed greater inter-sample dispersion. **(C)** Volcano plot showing differentially expressed genes (DEGs) between the DNP and Mock groups. **(D)** Volcano plot showing DEGs between the PACAP-38 and DNP groups. **(E**–**F)** Gene set enrichment analysis (GSEA) of the DNP vs. Mock (E) and PACAP-38 vs. DNP (F) comparisons. **(G**–**I)** Gene Ontology (GO) enrichment analysis of DEGs between the DNP and Mock groups for cellular component (CC), biological process (BP), and molecular function (MF), respectively. **(J**–**L)** GO enrichment analysis of DEGs between the PACAP-38 and DNP groups for CC, BP, and MF, respectively.

**Figure:**
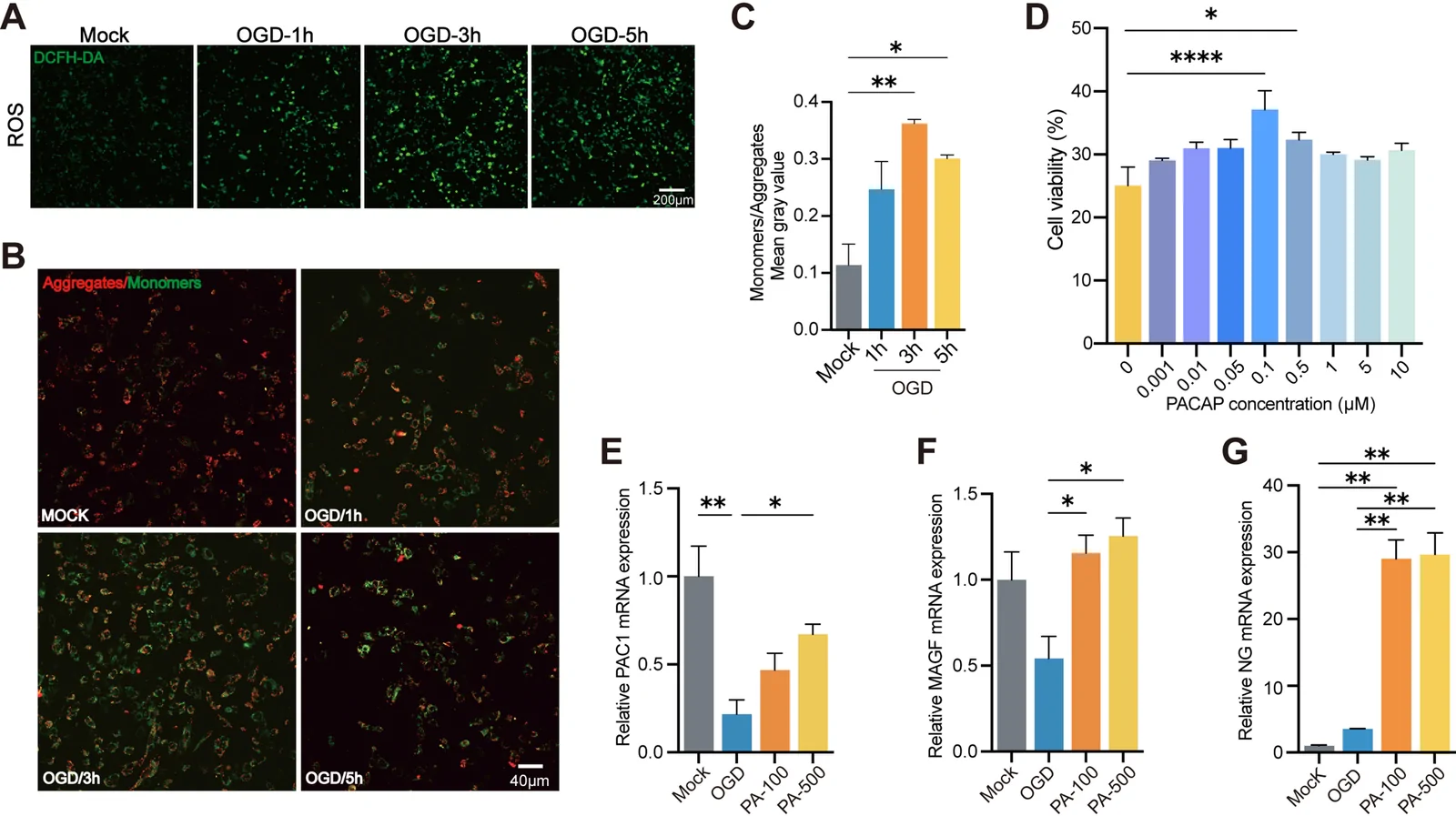
Fig. S3**Optimization of OGD duration and preliminary characterization of PACAP-38 responses in SH-SY5Y cells.** **(A)** Representative fluorescence images showing intracellular ROS levels in SH-SY5Y cells following 1, 3, and 5 h of OGD. **(B)** Representative JC-1 fluorescence images showing mitochondrial membrane potential in SH-SY5Y cells following 1, 3, and 5 h of OGD. **(C)** Quantification of the JC-1 monomer/aggregate fluorescence ratio. The ratio was significantly elevated at 3 h and 5 h of OGD relative to the Mock control, with the highest value observed at 3 h; therefore, 3 h OGD was selected for subsequent experiments. **(D)** Cell viability measured by CCK-8 assay in SH-SY5Y cells exposed to 3 h of OGD and treated with different concentrations of PACAP-38. PACAP-38 at 0.1 μM and 0.5 μM significantly attenuated the OGD-induced reduction in cell viability. **(E**–**G)** Relative mRNA expression levels of (E) PAC1, (F) MAGF, and (G) NG in SH-SY5Y cells treated with PACAP-38 under OGD conditions.

**Figure:**
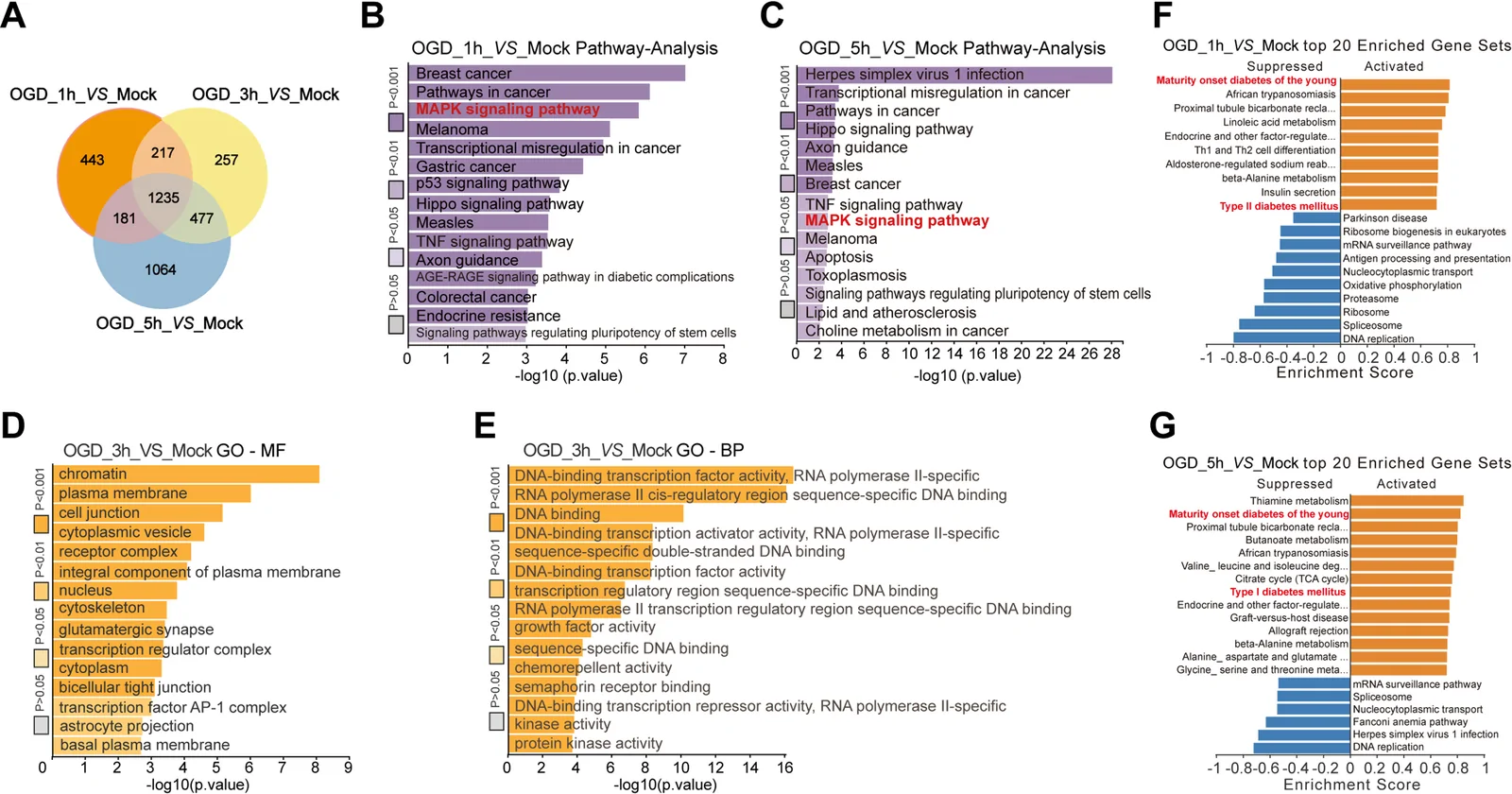
Fig. S4**Supplementary transcriptomic analyses of SH-SY5Y cells at different durations of OGD.** **(A)** Venn diagram showing the overlap of differentially expressed genes (DEGs) identified at 1, 3, and 5 h of OGD. DEGs were defined using the criteria of adjusted P < 0.05 and |log_2_FC| > 1. **(B–C)** Kyoto Encyclopedia of Genes and Genomes (KEGG) pathway enrichment analysis of DEGs identified at (B) 1 h and (C) 5 h of OGD. **(D**–**E)** Gene Ontology (GO) enrichment analysis of DEGs identified after 3 h OGD, categorized by (D) molecular function (MF) and (E) biological process (BP). **(F**–**G)** Gene set enrichment analysis (GSEA) of transcriptomic profiles at (F) 1 h and (G) 5 h of OGD.
